# Optimized DNA extraction from neonatal dried blood spots: application in methylome profiling

**DOI:** 10.1186/1472-6750-14-60

**Published:** 2014-07-01

**Authors:** Akram Ghantous, Richard Saffery, Marie-Pierre Cros, Anne-Louise Ponsonby, Steven Hirschfeld, Carol Kasten, Terence Dwyer, Zdenko Herceg, Hector Hernandez-Vargas

**Affiliations:** 1 Epigenetics Group, International Agency for Research on Cancer (IARC), 150 rue Albert-Thomas, 69008 Lyon, France; 2 Cancer and Disease Epigenetics, Murdoch Childrens Research Institute Royal Children's Hospital, Flemington Road Parkville, Victoria 3052, Australia; 3 Environmental & Genetic Epidemiology Research, Murdoch Children’s Research Institute Royal Children’s Hospital, Flemington Road Parkville, Victoria 3052, Australia; 4 Menzies Research Institute, University of Tasmania, Hobart TAS 7000, Australia; 5 Department of Health and Human Services, National Children’s Study, Eunice Kennedy Shriver National Institute of Child Health and Human Development, National Institutes of Health U.S., 6100 Executive Boulevard, Room 3A01, Bethesda, MD 20892, USA; 6 Chair, Steering Committtee, International Childhood Cancer Cohort Consortium (I4C

**Keywords:** Blood spot, DNA extraction, Epigenetics, Methylome, HM450, Pyrosequencing, Whole bisulfitome amplification, QIAamp, GenSolve, NucleoSpin

## Abstract

**Background:**

Neonatal dried blood spots (DBS) represent an inexpensive method for long-term biobanking worldwide and are considered gold mines for research for several human diseases, including those of metabolic, infectious, genetic and epigenetic origin. However, the utility of DBS is restricted by the limited amount and quality of extractable biomolecules (including DNA), especially for genome wide profiling. Degradation of DNA in DBS often occurs during storage and extraction. Moreover, amplifying small quantities of DNA often leads to a bias in subsequent data, particularly in methylome profiles. Thus it is important to develop methodologies that maximize both the yield and quality of DNA from DBS for downstream analyses.

**Results:**

Using combinations of in-house-derived and modified commercial extraction kits, we developed a robust and efficient protocol, compatible with methylome studies, many of which require stringent bisulfite conversion steps. Several parameters were tested in a step-wise manner, including blood extraction, cell lysis, protein digestion, and DNA precipitation, purification and elution. DNA quality was assessed based on spectrophotometric measurements, DNA detectability by PCR, and DNA integrity by gel electrophoresis and bioanalyzer analyses. Genome scale Infinium HumanMethylation450 and locus-specific pyrosequencing data generated using the refined DBS extraction protocol were of high quality, reproducible and consistent.

**Conclusions:**

This study may prove useful to meet the increased demand for research on prenatal, particularly epigenetic, origins of human diseases and for newborn screening programs, all of which are often based on DNA extracted from DBS.

## Background

Epigenetic mechanisms, such as DNA methylation, have been suggested as possible causal pathways linking environmental exposure to disease. Many of these studies depend on the epigenome-wide analysis of prospectively collected samples, in the context of large human cohorts. As epigenome-wide technologies are becoming available, the use of such cohort studies will provide large amounts of information in the coming years. Due to the general lack of biospecimen collection in observational human studies, many of these cohorts rely on the use of dried blood spots (DBS) obtained soon after birth as the main source of biological information [1].

The use of filter paper for blood collection and analysis was implemented as early as the 1960s by Guthrie et al. using dried-blood samples for newborn phenylketonuria detection [2]. “Guthrie cards” are widely used in many types of tests, including chemical, serological, and genetic applications [3]. More recently, Flinders Technology Associates chemically treated filter papers (FTA cards) were specifically developed for DNA/RNA analyses [4]. These chemically treated cards allow long-term storage of DNA at room temperature and are impregnated with denaturants that guard against oxidation, nuclease and ultraviolet damage, and both bacterial and fungal degradation.

Neonatal DBS are routinely collected in many countries and represent a cost-effective tool to store precious biological specimens for subsequent studies. However, reliable profiling the DNA methylome in DBS has proven to be technically challenging, particularly because such techniques require stringent bisulfite preprocessing that can degrade DNA [5]. Other limitations of their use include the variable degradation of DNA due to storage and extraction, the usually small amounts of DNA that can be obtained (typical blood spots are between 6 and 10 mm in diameter), and the identification of technical artifacts potentially associated with long term storage [6].

Recently, there has been increasing interest in the use of DBS in DNA methylome analyses, using Methylated DNA Immunoprecipitaion combined with sequencing (MeDIP-seq) [7], Methyl-CpG Binding Domain (MBD) protein-enrichment combined with sequencing (MBD-seq) [8], and Infinium (Illumina) bead arrays [7,9-11]. The last version of Illumina’s bead array, Infinium HumanMethylation450 (HM450) Beadchip, is cost-effective, requires DNA amounts as low as 300 ng, enables the detection and quantitation of DNA methylation levels at 486,685 CpG sites across the genome and represents one of the most comprehensive microarray methods to date for investigating the methylome [12]. Three reports have addressed the utility of HM450 on DBS [7,10,11]. The first report validated the use and the high correlation between two different methylomic platforms on DBS DNA: HM450 and MeDIP-seq [7]. The second one generated good quality methylome-wide data from DBS, as compared to their matched frozen buffy coat [10]. The third one used DBS-based HM450 analyses to study the epigenetic effects of gestational age, as recently demonstrated by our group [11]. However, none of these studies analyzed methods for optimized DNA extraction and quality verification from DBS, both of which represent major upstream steps in the pipeline for DBS-based research, including epigenetics.

In this report, we tested and developed a range of DNA extraction methods from neonatal FTA cards, individually or in combinations. We incorporated or modified protocol steps that were crucial to increase the DNA yield and quality from DBS, and additionally tested their efficiency on Guthrie cards. Moreover, we suggest an optimal protocol for both, pyrosequencing- and HM450-based, methylation studies. This work could prove useful in meeting the increased demand for research on prenatal origins of human diseases and for newborn screening programs.

## Results

### Optimization of Phases I and II in the DNA extraction protocols

**Figure 1:**
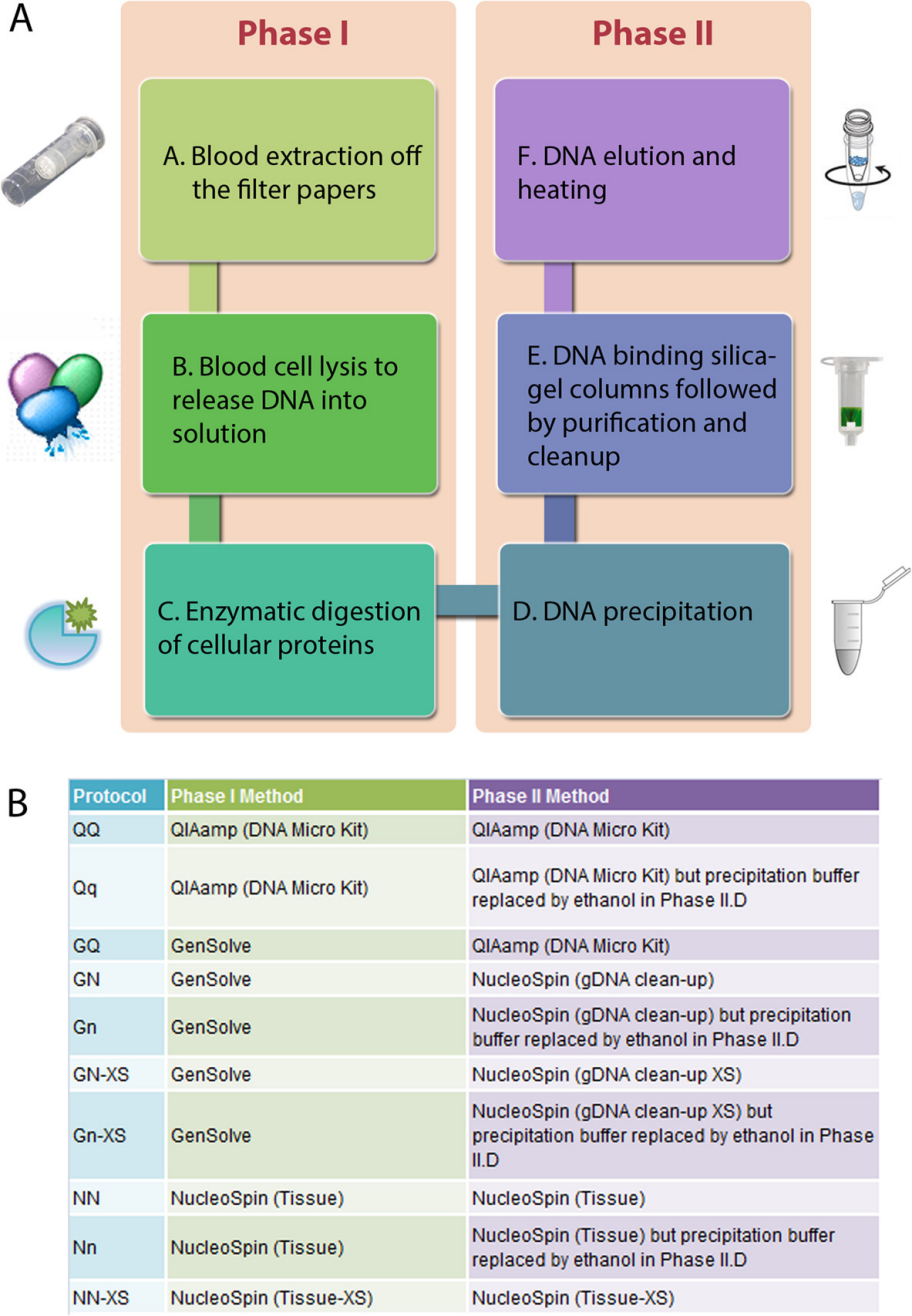
**Phases and classification of protocols used to extract DNA from DBS.** Two sequential phases, each encompassing three steps, are outlined **(A)** and were optimized in the different protocols or method combinations **(B)** used to extract DNA from DBS. A spin basket is shown next to Phase I.A and consists of a tube with an embedded perforated basket used to separate blood solutions from the filter papers from which they were extracted. A silica-gel column with a funnel-shape design is shown next to Phase II.E and often used to elute small volumes (5–30 μl), as manufactured by Macherey-Nagel and supplied with the extra-small (XS) versions of NucleoSpin kits **(B)**.

Limited quantity and quality are important drawbacks in the use of DNA obtained from DBS, particularly for epigenome-wide studies. Initially, we ruled out the possibility of using whole bisulfitome amplification (WGA) after confirming the introduction of biases, mostly in the middle range of DNA methylation levels (Additional file 1), further confirming the recently reported finding by Bundo et al. [13]. Then, to systematically optimize DNA extraction from DBS, we divided the different steps of this process into two phases (Figure 1). Critical steps in Phase I included blood extraction off the filter papers, cell lysis and protease digestion (Figure 1A, left panel). Phase II included DNA precipitation, purification and elution (Figure 1A, right panel).

We have previously tested several genomic DNA extraction methods on DBS, including resin-based, lysis-based and magnetic bead-based [5]. Lysis- and bead-based methods were the best, but the latter is not suitable for beadchip methylation profiling, so it was not considered in this study [9]. Among lysis-based methods, several commercially available kits, including QIAamp DNA Micro Kit, GenSolve and NucleoSpin, have been shown to be efficient for DBS DNA extraction [10,14,15]. Therefore, we selected these three kits to optimize the two phases of DNA extraction. This optimization involved the combination of the different kits and modifications in several steps of the two protocol phases (Figure 1B) (described in detail in Additional file 2). A combination of GenSolve reagents in Phase I and QIAamp reagents in Phase II (referred to as GQ method) was set as a reference protocol to which other tested methods were compared (Tables 1, 2, 3, 4, 5 and 6). In all pairwise comparisons, two DBS punches from the same DBS were used, and assessment of quantity and quality were initially done using Nanodrop (with 260/280 and 260/230 spectrophotometric ratios as a measure of quality) (Tables 1, 2, 3, 4 and 5). These DBS were obtained from the National Children Study (NCS), USA, and were FTA-type, which preserves well DNA relative to other types of neonatal cards (Methods), hence, allowing comparisons across a wide range of DNA extraction protocols.

**Table 1:** Combinations of Gensolve and Qiagen protocols for DNA extraction from DBS: GQ, QQ and Qq methods

						**65°C, 65 min**		
	**Sample code**	**DNA (ng/μl)**	**DNA (ng)**	**260/280**	**260/230**	**260/280**	**260/230**	**Protocol**
	NCS 11a	21.9	944	2.03	1.04	1.89	1.02	GQ
	NCS 11b	3.4	147	1.55	0.19	1.35	0.29	QQ
	NCS 12a	19.1	802	1.91	1.19	1.90	1.18	GQ
	NCS 12b	6.1	257	1.58	0.36	1.73	0.37	QQ
	NCS 13a	8.8	376	1.82	0.68	2.01	0.67	GQ
	NCS 13b	2.9	120	2.48	0.25	2.00	0.21	QQ
Precipitation buffer in QQ changed to ethanol, leading to protocol Qq	NCS 8a	13.9	596	1.88	0.73	1.74	0.79	GQ
	NCS 8b	32.1	1379	1.25	0.93	1.28	0.98	Qq
	NCS 9a	11.2	493	2.06	0.72	1.93	0.75	GQ
	NCS 9b	27.4	1178	1.22	0.75	1.19	0.76	Qq
	NCS 10a	12.0	502	2.07	0.88	2.01	0.92	GQ
	NCS 10b	31.9	1371	1.47	0.99	1.44	1.01	Qq
	NCS 4-1a	15.3	644	2.10	0.83	1.96	0.89	GQ
	NCS 4-1b	26.1	1124	0.74	0.58	0.73	0.60	Qq
	NCS 4-2a	24.7	1062	1.97	1.05	1.96	1.06	GQ
	NCS 4-2b	49.9	2048	0.85	0.73	0.87	0.76	Qq
	NCS 4-3a	28.5	1225	2.08	1.22	1.87	1.15	GQ
	NCS 4-3b	46.9	1969	1.50	1.23	1.50	1.21	Qq

Two punches, each having 9 mm diameter, were analyzed per DBS. Punches labeled “a” were tested with GQ while their matched pairs, labeled “b”, were tested with QQ or Qq. When the DNA precipitation buffer in QQ was changed to ethanol, the resultant protocol was termed Qq. Average eluate volume by GQ, QQ or Qq was 42 μl. Data represent averages of 2–4 readings per sample.

**Table 2:** **Combinations of GenSolve, Qiagen and NucleoSpin protocols for DNA extraction from DBS: GQ *versus *** **GN and Gn methods**

						**65°C, 65 min**		
	**Sample code**	**DNA (ng/μl)**	**DNA (ng)**	**260/280**	**260/230**	**260/280**	**260/230**	**Protocol**
	NCS 31a	5.77	242	1.70	0.42	1.55	0.41	GQ
	NCS 31b	8.87	426	1.81	0.95	1.54	0.71	GN
	NCS 30a	12.8	538	1.88	0.79	1.76	0.76	GQ
	NCS 30b	24.4	1171	1.84	1.22	1.90	1.51	GN
	NCS 29a	20.7	828	1.85	1.13	1.76	1.00	GQ
	NCS 29b	23.1	1063	1.93	1.38	1.85	1.37	GN
Precipitation buffer in GN changed to ethanol, leading to protocol Gn	NCS 22a	9.85	394	1.81	0.50	1.79	0.48	GQ
	NCS 22b	8.00	368	1.90	0.83	1.79	0.75	Gn
	NCS 21a	24.3	972	1.83	0.84	1.83	0.90	GQ
	NCS 21b	27.7	1274	1.64	0.54	1.83	0.61	Gn
	NCS 20a	15.0	600	1.78	0.56	1.77	0.60	GQ
	NCS 20b	14.4	662	1.70	0.76	1.74	0.89	Gn

Two punches, each having 9 mm diameter, were analyzed per DBS. Punches labeled “a” were tested with GQ while their matched pairs, labeled “b”, were tested with GN. When the DNA precipitation buffer in GN was changed to ethanol, the resultant protocol was termed Gn. Average eluate volumes by GQ and GN/Gn were 42 μl and 47 μl, respectively. Data represent averages of 2–4 readings per sample.

**Table 3:** **Combinations of GenSolve, Qiagen and NucleoSpin protocols for DNA extraction from DBS: GQ *versus *** **GN-XS and Gn-XS methods**

						**65°C, 65 min**		
	**Sample code**	**DNA (ng/μl)**	**DNA (ng)**	**260/280**	**260/230**	**260/280**	**260/230**	**Protocol**
	NCS 28a	20.3	873	1.84	0.95	1.82	0.97	GQ
	NCS 28b	37	1813	1.87	0.11	1.78	0.13	GN-XS
	NCS 27a	19.4	834	1.85	0.85	1.80	0.90	GQ
	NCS 27b	27.9	1339	2.19	0.61	2.26	0.61	GN-XS
	NCS 26a	19.7	847	1.83	0.97	1.74	0.93	GQ
	NCS 26b	23.7	1161	1.66	0.79	1.77	0.80	GN-XS
	NCS 25a	16.3	701	1.9	1.07	1.79	1.07	GQ
	NCS 25b	21.7	1042	1.83	0.34	1.96	0.28	GN-XS
	NCS 24a	11.3	486	1.82	0.78	1.79	0.82	GQ
	NCS 24b	21.6	1037	1.53	0.08	1.48	0.07	GN-XS
	NCS 23a	11.9	512	1.18	0.49	1.27	0.54	GQ
	NCS 23b	11.4	547	1.44	0.50	1.52	0.42	GN-XS
Precipitation buffer in GN-XS changed to ethanol, leading to protocol Gn-XS	NCS 19a	30.1	1174	1.53	0.71	1.57	0.81	GQ
	NCS 19b	15.1	725	1.49	0.65	1.50	0.70	Gn-XS
	NCS 18a	12.2	488	1.81	0.62	1.78	0.64	GQ
	NCS 18b	20.4	979	1.30	0.64	1.44	0.72	Gn-XS
	NCS 17a	59	2360	1.80	1.37	1.91	1.56	GQ
	NCS 17b	42.6	2130	1.65	0.53	1.75	0.57	Gn-XS
Washing volume and frequency in GN-XS increased	NCS 16a	16.8	823	1.71	0.76	1.80	0.78	GQ
	NCS 16b	13.1	707	1.43	0.21	1.55	0.22	GN-XS
	NCS 15a	27.1	1382	1.76	1.04	1.79	0.97	GQ
	NCS 15b	12.6	668	7.83	0.39	Error *	0.45	GN-XS
	NCS 14a	30.3	1545	1.77	1.01	1.83	1.07	GQ
	NCS 14b	22.8	1208	2.37	0.60	2.60	0.61	GN-XS

Two punches, each having 9 mm diameter, were analyzed per DBS. Punches labeled “a” were tested with GQ while their matched pairs, labeled “b”, were tested with GN-XS. When the DNA precipitation buffer in GN-XS was changed to ethanol, the resultant protocol was termed Gn-XS. For NCS 16b, 15a and 14b, the washing volume was increased from 100 to 500 μl, and washing was performed twice instead of once. Average eluate volumes by GQ and GN-XS/Gn-XS were 42 μl and 48 μl, respectively. Data represent averages of 2–4 readings per sample. ^*^ The error represents values out of range.

**Table 4:** **Combinations of GenSolve, Qiagen and NucleoSpin protocols for DNA extraction from DBS: GQ *versus *** **NN and Nn methods**

						**65°C, 65 min**		
	**Sample code**	**DNA (ng/μl)**	**DNA (ng)**	**260/280**	**260/230**	**260/280**	**260/230**	**Protocol**
	NCS 1a	25.3	1062	1.84	1.15	1.88	1.18	GQ
	NCS 1b	40.0	1802	1.82	1.84	1.90	1.90	NN
	NCS 2a	28.8	1209	1.87	1.27	1.89	1.33	GQ
	NCS 2b	47.3	2127	1.89	2.00	1.90	1.91	NN
	NCS 3a	15.2	638	2.02	1.09	1.93	1.15	GQ
	NCS 3b	27.6	1271	1.82	1.59	1.91	1.61	NN
	NCS 4-4a	23.0	987	1.93	0.93	1.96	1.03	GQ
	NCS 4-4b	30.0	1348	1.85	1.72	1.90	1.81	NN
	NCS 4-5a	33.4	1435	1.82	1.01	1.83	1.02	GQ
	NCS 4-5b	35.4	1593	1.98	1.94	1.87	1.76	NN
	NCS 4-6a	19.9	856	2.01	1.14	1.97	1.10	GQ
	NCS 4-6b	33.9	1525	1.93	1.85	1.89	1.82	NN
Precipitation buffer in NN changed to ethanol, leading to protocol Nn	NCS 4-7a	18.0	774	1.76	0.87	1.86	0.84	GQ
	NCS 4-7b	25.7	1155	1.93	1.37	1.81	1.35	Nn
	NCS 4-8a	14.7	617	1.99	0.90	1.94	0.91	GQ
	NCS 4-8b	24.6	1105	1.92	1.33	1.85	1.42	Nn
	NCS 4-9a	26.1	1121	1.90	1.20	1.94	1.26	GQ
	NCS 4-9b	39.0	1754	1.95	1.89	1.88	1.77	Nn

Two punches, each having 9 mm diameter, were analyzed per DBS. Punches labeled “a” were tested with GQ while their matched pairs, labeled “b”, were tested with NN. When the DNA precipitation buffer in NN was changed to ethanol, the resultant protocol was termed Nn. Average eluate volumes by GQ and NN/Nn were 42 μl and 45 μl, respectively. Data represent averages of 2–4 readings per sample.

**Table 5:** **Combinations of GenSolve, Qiagen and NucleoSpin protocols for DNA extraction from DBS: GQ *versus *** **NN-XS methods**

					**65°C, 65 min**		
**Sample code**	**DNA (ng/μl)**	**DNA (ng)**	**260/280**	**260/230**	**260/280**	**260/230**	**Protocol**
NCS 4a	17.5	734	1.98	0.96	1.84	0.98	GQ
NCS 4b	17.3	833	1.72	0.84	1.65	0.78	NN-XS
NCS 5a	17.6	741	1.82	1.05	1.81	1.07	GQ
NCS 5b	12.8	614	1.61	1.02	1.73	1.07	NN-XS
NCS 6a	8.2	328	2.02	0.81	1.79	0.76	GQ
NCS 6b	15.7	756	1.70	0.89	1.70	0.86	NN-XS
NCS 4-10a	22.2	956	1.95	1.21	2.02	1.19	GQ
NCS 4-10b	13.5	660	1.84	1.10	1.85	1.08	NN-XS
NCS 4-11a	19.1	822	1.60	0.85	1.71	0.88	GQ
NCS 4-11b	12.6	618	1.60	1.00	1.58	1.11	NN-XS
NCS 4-12a	10.4	447	1.54	0.74	1.76	0.74	GQ
NCS 4-12b	13.1	657	1.55	0.76	1.70	0.74	NN-XS

Two punches, each having 9 mm diameter, were analyzed per DBS. Punches labeled “a” were tested with GQ while their matched pairs, labeled “b”, were tested with NN-XS. Average eluate volumes by GQ and NN-XS were 42 μl and 49 μl, respectively. Data represent averages of 2–4 readings per sample.

**Table 6:** Cross-comparisons of DNA quantity and quality parameters among the different tested DNA extraction protocols

**Protocol**	**DNA quantity (ng)**^**a**^	**260/280 ratio (after heating)**^**b**^	**260/230 ratio (after heating)**^**c**^	**DNA integrity**^**d**^	**Detectability by PCR**
***GQ***	808 ± 376 ng	1.83 ± 0.14	0.92 ± 0.23	Peak intensity > 1 Kbp (25/32)	Detectable (42/42)
***QQ***	Lower (3/3) 175 ± 73 ng	Similar (2/3); Worse (1/3)	Lower (3/3)	NA^e^	**Similar (3/3)**
***Qq***	**Higher (6/6) by 2.0 ×**	Worse (6/6) 0.89 ± 0.22	**Similar (6/6)**	**Similar (4/6); Better (1/6); Worse (1/6)**	**Similar (6/6)**
***GN*** *or* ***Gn***	**Higher (4/6) by 1.5 ×; Similar (2/6)**	**Similar (6/6)**	**Similar (4/6); Higher (2/6)**	Worse (4/4)	**Similar (6/6)**
***GN-XS*** *or* ***Gn-XS***	**Higher (6/9) by 1.7 ×; Similar (2/9); Lower (1/9) by 0.4 ×**	Similar (6/9); Worse (3/9)	Similar (5/9); Lower (4/9)	Worse (6/6)	Similar (9/9)
***NN*** *or* ***Nn***	**Higher (8/9) by 1.7 ×; Similar (1/9)**	**Similar (9/9)**	**Higher (9/9) 1.71 ± 0.20**	Worse (6/6)	**Similar (9/9)**
***NN-XS***	Similar (2/6); Higher (2/6) by 1.8 ×; Lower (2/6) by 0.3 ×	**Similar (5/6); Worse (1/6)**	**Similar (6/6)**	Worse (3/5); Similar (1/5); Better (1/5)	**Similar (6/6)**

The GQ protocol is set as the reference protocol above all the other protocols to which it is compared. The DNA quantity or quality parameters of the other protocols are described always in comparison to GQ. DNA yields of QQ were significantly lower than GQ (p < 0.05), those of Qq, GN, Gn, NN and Nn were significantly higher than GQ (p < 0.05), and those of GN-XS, Gn-XS and NN-XS were not different from GQ (p > 0.05), as compared by paired t-test. For each DNA parameter described, the counts of hits over the total number of DBS analyzed for that parameter are indicated between parentheses. Table cells highlighted in bold represent protocol performance that is at least as good as that of GQ.

^a^Quantities showing less than 20% change from GQ were considered ‘similar’ to GQ. This threshold exceeds the 15.7% average increase in DNA quantities observed between GQ technical replicates, with an average coefficient of variation of 12.3% (n = 6 pairs of replicates).

^b^In protocol pairwise comparisons, 260/280 ratios that were considered ‘similar’ were either both within the optimal 1.7-2.0 range or both outside this range. Otherwise, the ratio outside the 1.7-2.0 range was considered ‘worse’ relative to that within.

^c^‘Lower’ and ‘higher’ indicate 260/230 ratio differences of at least 0.30 absorbance units below or above GQ ratios, respectively; otherwise, ratios were considered ‘similar’. The following guidelines were adopted for the 260/230 ratio: optimal and indicating pure DNA if higher than 2.0, acceptable if between 1.5-2.0, and tolerated if between 1.0-1.49 (Macherey-Nagel GmbH & co. KG, reference 740230).

^d^DNA integrity refers to DNA size range and level of degradation, as assessed by gel electrophoresis. Ten samples were also reassessed by bioanalyzer, showing similar relative comparisons to GQ. Every DBS, in which both punches of the tested protocol pairs exhibited high DNA degradation (size range below 1 Kbp), was excluded from the pairwise comparisons of protocols.

^e^NA: Not Applicable due to limited quantities of extractable DNA.

DNA yield and quality were consistently better for the reference GQ protocol when compared to Qiagen protocol (QQ) (Table 1, p < 0.05). Although DNA yield was drastically increased when ethanol was used in the Qiagen precipitation step (Qq, p < 0.001), DNA quality was still suboptimal compared to GQ, as assessed by nanodrop (Table 1). In contrast, the combination of Gensolve and NucleoSpin (GN and Gn) increased the DNA yield while preserving DNA quality, regardless of the use of ethanol in the precipitation step (Table 2, p < 0.05). A similar improvement was observed when using NucleoSpin kit in both phases of DNA extraction (NN and Nn) (Table 4, p < 0.001). The extra-small (XS) versions of NucleoSpin, with column designs specific for low elution volumes, did not consistently improve DNA quantity or quality, whether combined or not with other kits (GN-XS, Gn-XS and NN-XS; Tables 3, 5 and 6), the DNA precipitation buffer changed to ethanol, or the washing volume and frequency increased (Table 3, p > 0.05).

### Cross-comparisons across the different tested DNA extraction protocols

**Figure 2:**
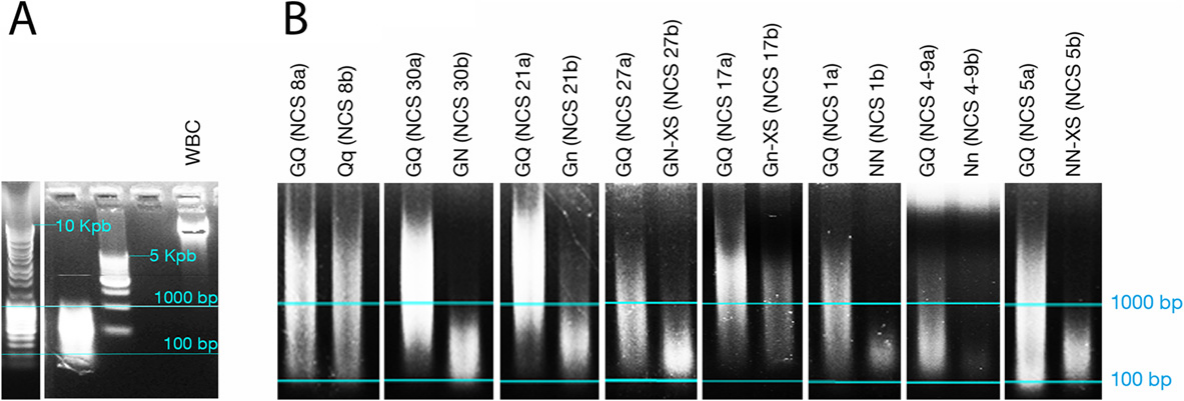
**DNA integrity and size range as assessed by agarose gel electrophoresis. (A)** DNA size markers used to estimate size ranges are shown in addition to genomic DNA that was isolated from white blood cells (WBC) and used as a positive control. **(B)** Representative DBS from each of the tested protocols are shown, except for protocol QQ in which DNA amounts were insufficient to be analyzed by gel electrophoresis. Eight different gel sections are shown and are derived from either the same gel or different gels. In each section, two punches from the same NCS spot were run on the same gel, with the first punch, labeled ‘a’, representing protocol GQ and the second punch, labeled ‘b’, representing another unique protocol from the tested set. The two blue lines, representing the 100 and 1000 base pair (bp) size ranges, were set according to the molecular size marker used in each section. The 1000 base pair limit is a minimum size range with useful applications in many genetic and epigenetic studies, including Illumina’s HM450 Beadchip array. The results of other DBS analyzed by gel electrophoresis or bioanalyzer are summarized in Table 6.

DNA quality parameters assessed earlier were based on DNA 260/280 and 260/230 spectrophotometric ratios. Two other important quality parameters are DNA detectability by PCR and DNA integrity and size range, which can be assessed by gel electrophoresis and bioanalyzer analyses. DNA from all tested protocols exhibited detectable PCR bands of a housekeeping gene, *GAPDH* (Table 6 and data not shown), hence indicating that the DNA is amplifiable for specific short regions. DNA isolated by the GQ method exhibited a smear-like profile by gel electrophoresis, with peak intensity often greater than 1 kilo base pair (Kbp) (Figure 2 and Table 6). Bioanalyzer smear analyses confirmed the DNA average size peak to be greater than 1 Kbp, with an average size ranging across samples between 4.9-9.7 Kbp (Table 6 and data not shown). Compared to GQ, all tested protocols often showed more DNA degradation, except for protocol Qq which usually exhibited similar DNA smear profiles (Figure 2 and Table 6).

Overall comparison of the tested DNA quantity and quality parameters across the protocols shows that the best two protocols that at least match GQ in most of the tested parameters are Qq and NN (or Nn) (Table 6). Relative to GQ, the only drawback of Qq is its 260/280 DNA ratios (Table 6). Qq 260/280 ratios were always out of range, indicating that the high Qq DNA quantity measurements recorded spectrophotometrically by nanodrop may not be accurate. However, quantification with Qubit, a fluorescent-based method, confirmed the yield in Qq to be 2.7× higher than in GQ (data not shown). The only drawback of NN (or Nn) relative to GQ was DNA integrity, with the DNA size ranges in NN or Nn being lower than in GQ (Table 6).

In conclusion, protocol GQ seems to be the most robust among the tested methods across all tested DNA quantity and quality parameters. Qq can be rather suitable for applications requiring larger DNA quantities from DBS, while maintaining large fragment sizes, but in which 260/280 ratios are not a necessity. On the other hand, protocols NN or Nn may be better suitable for applications requiring larger DNA quantities from DBS, relative to GQ, while maintaining optimal 260/280 and better 260/230 ratios, but in which large DNA fragment sizes are not a requirement.

Of note, when GQ was tested on DBS (Guthrie cards, Whatman 903) from the Tasmanian Infant Health Survey (TIHS), Australia, dating more than 20 years old, an average of 66 ± 15 ng (n = 3) of DNA could be extracted per two punches, each being 1 mm in diameter, with a mean 260/280 ratio of 1.66 ± 0.02; these DNA quantities are equivalent to 42.0 ng/mm^2^ for TIHS, compared to 12.7 ng/mm^2^ for NCS samples.

### Performance of DNA extracted from DBS using methylome-wide analysis

#### Methylation probe call index

In order to assess the performance of DNA extracted from DBS in HM450 methylome-wide analyses, we used the DNA extracted by GQ, being the most robust protocol. DNA from two sample pairs, each representing two punches (serving as technical replicates) from the same DBS, were analyzed by HM450. In addition, DBS pairs were compared to reference DNA from neonatal blood or cell lines (Table 7). In all tested samples, whether originating from neonatal blood, DBS or cell lines, more than 99% of the 485,577 HM450 individual probes were detected, using the commonly accepted quality control detection p-value of 0.01, hence, indicating high quality data. The average beta-values were similar between the technical replicates NCS 37a and 37b (approximately 0.47 for either) and between the technical replicates NCS 38a and 38b (approximately 0.42 for either) (Table 7).

**Table 7:** Methylation quality control probe evaluation

		**Probe**		**Beta-value**		
	**Sample**	**Number of CpGs detected with p < 0.01**	**Percentage of CpGs detected with p < 0.01**	**Average**	**Minimum**	**Maximum**
**Neonatal Blood DNA**	NB 1672	485405	99.96	0.4886	0.0012	0.9929
	NB 1597	485392	99.96	0.4729	0.0006	0.9947
	NB 1842	485358	99.95	0.4911	0.0009	0.9940
	NB 1645	485119	99.91	0.4704	0.0011	0.9914
**DBS DNA**	NCS 37a	484990	99.88	0.4712	0.0038	0.9953
	NCS 37b	484946	99.87	0.4719	0.0045	0.9916
	NCS 38a	483897	99.65	0.4226	0.0005	0.9942
	NCS 38b	482519	99.37	0.4240	0.0001	0.9935
**Cell Line DNA**	Cell Line 1	485124	99.91	0.4748	0.0022	0.9926
	Cell Line 2	485175	99.92	0.4813	0.0032	0.9920
	Cell Line 3	485342	99.95	0.4738	0.0006	0.9926
	Cell Line 4	485272	99.94	0.4743	0.0021	0.9934

Three sources of DNA are used in HM450 array: neonatal blood (NB), DBS and cell lines. NB and cell line DNA is of good genomic quality and serves as technical reference. NB provides DNA from the same tissue origin as DBS, being blood. The cell line DNA is of hepatic origin. The technical pairs are represented by two punches from each of two blood spots and are labeled as ‘NCS 37a and 37b’ and ‘NCS 38a and 38b’, respectively. Probe p-value was set to 0.01. The percentage of detected probes (p < 0.01) represents the proportion out of the total of 485,577 probes on the HM450 array. The average, minimum and maximum beta-values with detection p < 0.01 are shown (background was not subtracted, so minimum beta-values are not exactly zeros).

#### Sample–dependent and –independent HM450 internal quality control probes

**Figure 3:**
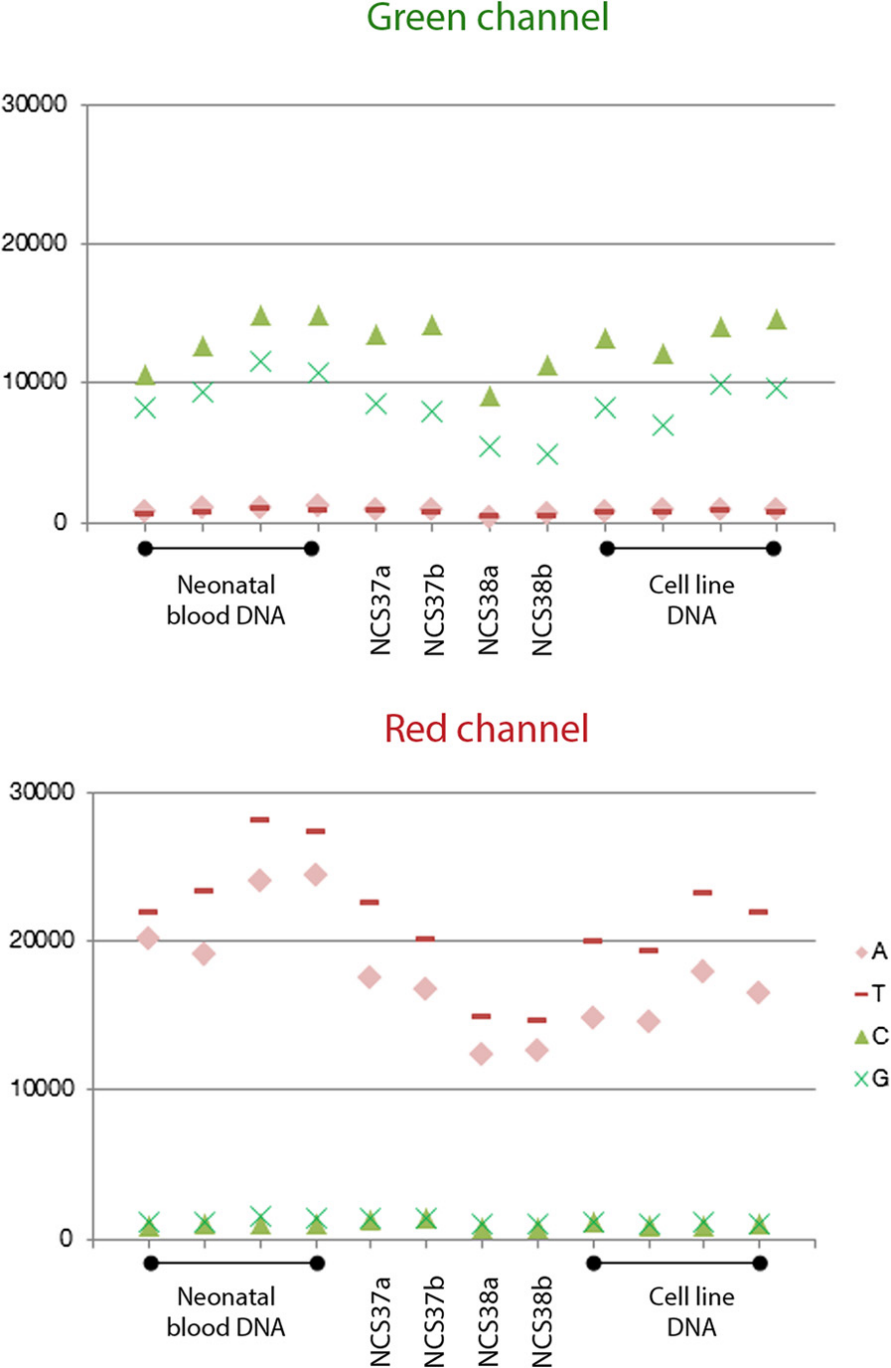
**HM450 QC plot using Non-polymorphic probes which assess overall performance.** In the green channel, background signals are shown in red and pink while positive signals in opaque and fluorescent green. In the red channel, background signals are shown in opaque and fluorescent green while positive signals in red and pink. One non-polymorphic control has been designed for each of the four nucleotides A, T, C, and G. Four DBS DNA samples are shown between four neonatal blood and four cell line DNA samples, in each of the two plots. The DBS samples represent two NCS spots, 37 and 38, each consisting of two tested punches labeled ‘a’ or ‘b’.

For sample and array quality, HM450 array includes 850 quality control (QC) probes. Fifteen QC probes are sample-independent and 835 QC probes are sample-dependent [12]. DNA from DBS, neonatal blood or cell lines passed the described HM450 QC (Additional file 3 and Figure 3). Background probes, wherever included, produced minimal signal (maximum limit is 1000 units, as recommended by Illumina Inc.), and the intended positive signals from the experimental QC probes were above background, for all of the three tested DNA sources (Additional file 3 and exemplified in Figure 3 using non-polymorphic probes, which are indicative of overall performance). In addition, performance of DBS samples was similar to that of subsets taken from reference neonatal blood and cell line samples (Additional file 4 and Figure 3). Bisulfite conversion efficiency for both, type I and II probes, was high for all tested samples (Additional file 4) and was confirmed by PCR using primers that are specific either to bisulfite converted or to non-modified *GAPDH* DNA regions (data not shown).

#### Differential methylation and clustering analyses using HM450 data

**Figure 4:**
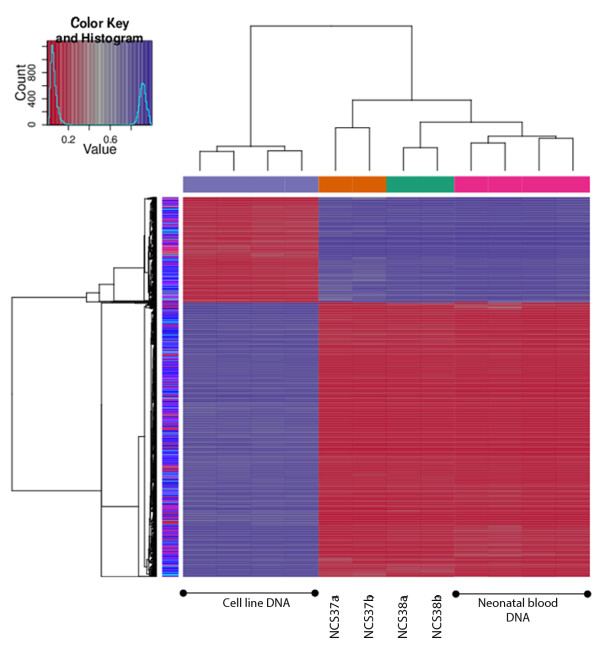
**Differential methylation and unsupervised clustering analysis of HM450 data from neonatal blood, DBS and cell line DNA.** Neonatal blood and cell line DNA samples are used as positive controls of good DNA quality for reference comparisons with DNA extracted from DBS. Neonatal blood and DBS are from different individuals. Four DBS DNA samples are shown between four different neonatal blood and four different cell line DNA samples. The DBS samples represent two NCS spots, 37 and 38, each consisting of two tested punches labeled ‘a’ or ‘b’. HM450 beta-values were clustered using Euclidean distance as the dissimilarity index. As shown in the color key, the red and blue signals represent relatively hypomethylated and hypermethylated regions, respectively.

Differential methylation of HM450 beta-values produced two major clusters (Figure 4). Cell line DNA samples formed one cluster while DBS and neonatal blood DNA formed another; this is expected because the cell lines used are of hepatic tissue origin while both, DBS and neonatal blood samples, are of blood tissue origin. Within the cluster of blood biospecimens, all four neonatal blood samples formed one sub-cluster, which was segregated away from the DBS sub-cluster. The two punches NCS 37a and 37b, representing the technical replicates from the same spot NCS 37, clustered together and away from the other two technical replicates, NCS 38a and 38b, which also clustered together (Figure 4). This further supports the observed higher correlations (p < 0.001, Steiger Z test) between technical duplicates punched from the same DBS (r^2^ range = 0.963-0.990) *versus* different DBS (r^2^ range = 0.949-0.951; Additional file 4A). When the analysis was limited to the top 1% of probes that showed the highest variance in M-values (transformed betas) across any of the four tested DBS, NCS_37a, NCS_37b, NCS_38a and NCS_38b, the correlation between replicates (r^2^ range = 0.850-0.972; p < 0.001, Pearson) become significantly higher and more predictive of replication (p < 0.001, Steiger Z test) than the correlation between non-replicates (r^2^ range = 0.365-0.371; p < 0.001, Pearson) (Additional file 4B). Similar observations were reported using Spearman correlations. Moreover, the frequency distributions of delta M-values (δM) between samples were centred at zero only between technical replicates (Additional file 4B). Hence, we can conclude that HM450 analyses using DBS DNA, extracted using the GQ protocol, is reproducible.

### Performance of DNA extracted from DBS using sequence-specific methylation analysis

**Figure 5:**
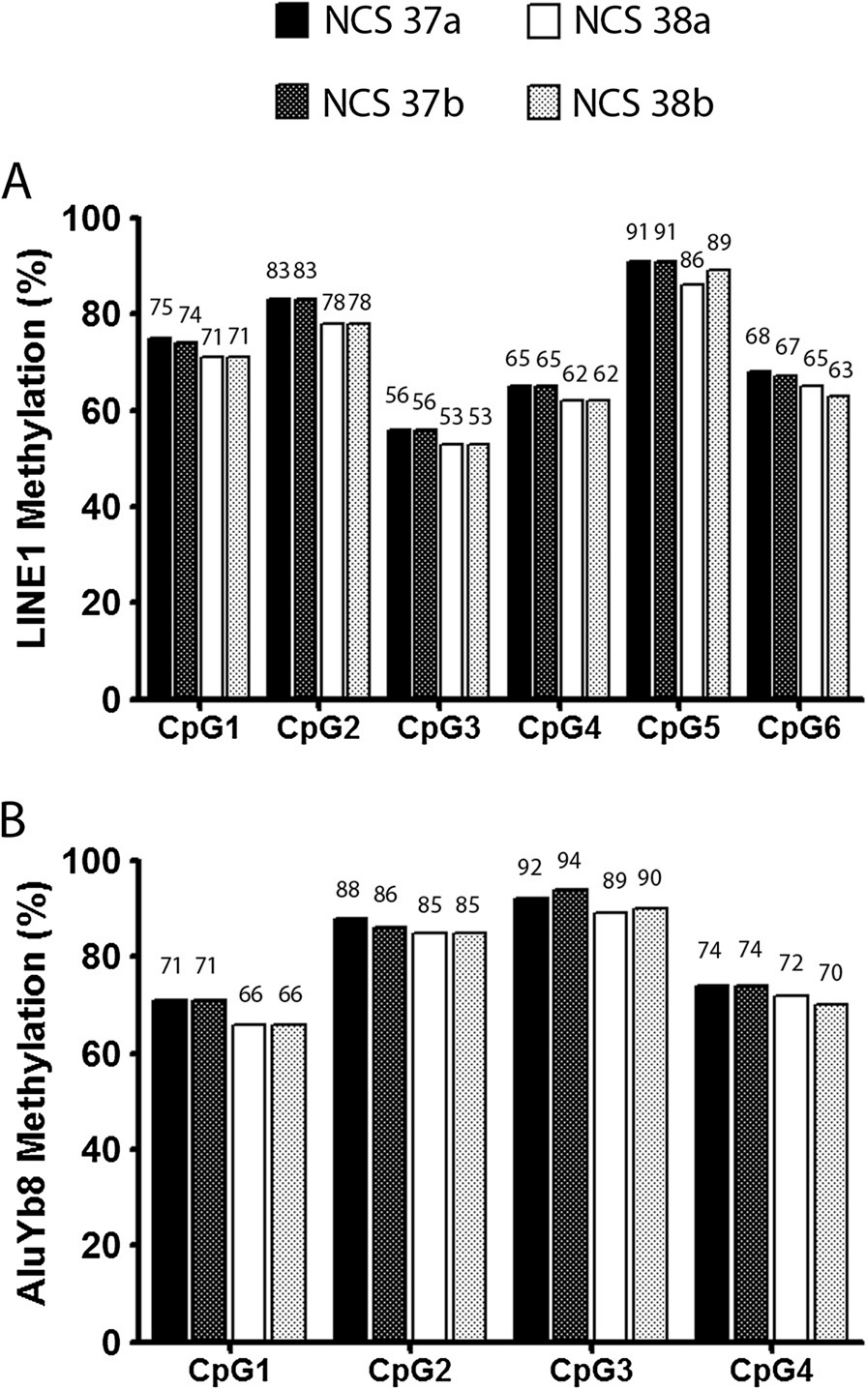
**Methylation analyses of *Line1 *and *AluYb8 *loci using bisulfite pyrosequencing.** The methylation levels of six and four CpG sites were analyzed for *Line1***(A)** and *AluYb8***(B)**, respectively, and are expressed as percent of the total number of CpGs analyzed for each individual CpG site. The DBS samples represent two NCS spots, 37 and 38, each consisting of two tested punches labeled ‘a’ or ‘b’.

The performance of DNA extracted from DBS by the GQ method was then tested using sequence-specific methylation analyses. For this purpose, we analyzed the methylation levels of several CpG sites in Line1 and AluYb8 sequences, both of which are proxy markers of global methylation, being transposable elements interspersed across the genome [16]. The technical replicates, NCS 37a and 37b, showed similar Line1 and AluYb8 methylation levels at each tested CpG site, and similar data were observed with the pair, NCS 38a and 38b (Figure 5A and B). These results show that inter-replicate variation in methylation levels is minimal using several CpG sites in two different loci, Line1 and AluYb8. Moreover, the observed difference in methylation levels between the pair NCS 37a and 37b *versus* NCS 38a and 38b was consistent at every single CpG tested and across both, Line1 and AluYb8 loci (p < 0.1 for CpG6 in Line1 and CpG3 in AluYb8 and p < 0.05 for all other CpGs; Mann–Whitney test) (Figure 5A and B). These results confirm that DNA extracted from DBS using GQ is suitable to detect small methylation differences in a consistent manner and with low inter-replicate variation.

## Discussion

DBS have become an increasingly important tool for diagnostic purposes and for epigenetic, genetic and epidemiological research. We have previously tested a range of commercially available DNA extraction kits for purifying genomic DNA from fresh and dried blood for downstream PCR and DNA methylation applications [5]. We found that genomic DNA extraction, using the ChargeSwitch Forensic DNA Purification kit (Invitrogen), with subsequent bisulfite modification, using the MethylEasy kit (Human Genetic Signatures), was best in yielding bisulfite-converted DNA of sufficient quantity and quality for downstream candidate-gene DNA methylation analyses, such as SEQUENOM MassArray EpiTYPER analysis [5]. However, DNA extraction with ChargeSwitch was recently shown to be not suitable for beadchip methylation profiling, leading to up to 16% loss of detectable probes in Infinum HumanMethylation27 (Illumina Inc.) arrays analysis [9]. In relation to the limited amounts of DNA extracted from DBS, a recent report also pointed to the biases introduced by whole bisulfitome amplification and the need for careful data interpretation [13], as we have also observed in this study.

In this work, we have systematically compared different DNA extraction methods from DBS by dissecting different phases of extraction and optimizing several steps within each phase, using commercial and in-house extraction protocols. For these purposes, we used a homogenous set of DBS samples, spotted on the same day and stored in a similar manner, to provide a common platform for cross-protocol comparisons. Moreover, this study emphasizes DNA extraction protocols that have particular utility in a recent technology for studying methylome-wide methylation, Infinium HM450, and sequence-specific methylation, by pyrosequencing. The use of DBS for diagnostic and research purposes is not new, but there is a lack of quality standards for optimizing DNA extraction. This study suggests different DNA extraction protocols, each having specific advantages tailored for specific applications. Protocol GQ does not extract the highest DNA yield, but provides DNA in quantities and qualities sufficient for HM450 methylome-wide and sequence-specific methylation analyses. With GQ, the 260/280 ratios are consistently optimal and the extracted DNA is less fragmented relative to other protocols. Protocol Qq, on the other hand, produces twice as much DNA as GQ and with similar DNA integrity. However, 260/280 ratios in Qq are unreliable and cannot be used for sample selection, particularly for expensive downstream applications. As for protocols NN or Nn, they extract 1.7 folds more DNA than GQ and show optimal ranges for both, 260/280 and 260/230 ratios. However, these protocols lead to more DNA fragmentation relative to GQ. This may represent a limitation for bead array-based assays and other DNA methylation assays where DNA integrity is a requirement. It should be noted though that 260/280 and 260/230 ratios should be treated with caution; for example, different contaminants can compensate for each other’s’ deviations, resulting in misleading optimal 260/280 ratios.

Because the type of purification column was identical in GQ and Qq, but different from those used in all the other methods, column nature (Phase II) could be the reason for the better DNA integrity in the two protocols. This is further supported by our results showing that in GQ, changing Phase II (includes column type) while maintaining Phase I, as in GN/n or GN/n-XS, compromises DNA integrity (Table 6). Changing the DNA precipitation buffer to ethanol reduces the need in some protocols to vigorously vortex to dissolve resultant precipitates but does not seem to enhance DNA integrity (Figure 2; compare GN *versus* Gn, GN-XS *versus* Gn-XS, and NN *versus* Nn). However, ethanol, as a precipitating buffer, was essential in some protocols to increase the DNA yield, as in Qq versus QQ (Tables 1 and 6).

Other studies have compared DNA extraction protocols from DBS, but irrespective of epigenetic applications [17-19]. It is difficult to compare DNA extraction protocols across different studies due to many reasons, such as differences in the structures of filter papers on which blood was soaked, the storage conditions and year-durations, and DNA quantifications methods used. However, these studies used QIAamp DNA Mini Kit (Qiagen) as a reference method, which is similar to the QQ protocol in this work, hence, allowing comparisons between our and their optimized methods. Sjoholm et al. reported that QIAamp DNA Mini Kit performed the best relative to EZNA (Omega Bio-Tek), Chelex 100 (AmershamBiosciences) and alkaline lysis (GenomiPhi DNA Amplification Kit, AmershamBiosciences) [19]. On the other hand, the in-house developed DNA extraction methods reported by Hue et al. and Hollegaard et al. yielded, by Nanodrop quantification, 3.3 and 2.5 fold more DNA, respectively than QIAamp DNA Mini Kit [18]. In comparison, the GQ protocol in our study yielded, also by Nanodrop quantification, on average 4.6 fold more DNA than matched QQ samples; in addition, protocols Qq, NN and Nn yielded at least 1.7 folds more DNA than matched GQ samples. Moreover, the in-house protocol by Hue et al. produced a low purity 260/280 average ratio (1.50) [18], while GQ, NN and Nn ratios were optimal in every tested sample. These findings support the good performance of our optimized methods relative to many other in-house and commercial DNA extraction protocols from DBS. Interestingly, one study reported a recent method suitable for performing scalable DNA extractions simultaneously from many DBS, but with less emphasis on DNA quality and yield comparisons across different methods [20]. The scale of our tested methods can be increased by implementing the QIAcube technology (Qiagen), and, with scalable designs, laser cutting of DBS punches would eliminate cross-contamination, as has been recently reported [21].

## Conclusion

This study arises from an international effort across several cohorts and working groups aiming to fulfill the need to systematize quality standards in DNA extraction and to increase the DNA yield using DBS, particularly with the advent of high-throughput epigenomic technology that require high quality and quantity of DNA. Given the emerging appreciation of DBS collected at birth as a valuable resource for epigenetic analyses prior to phenotypic onset, our optimized methods for DNA extraction with application in methylation analyses have great potential for diagnostic and research purposes.

## Methods

### Sample overview

DBS that were used to perform the comparisons across the DNA extraction protocols were obtained from NCS, USA, and have been spotted on Flinders Technology Associates (FTA) mini cards on the same day and dried in air-sealed containers for approximately two years at room temperature. Guthrie cards (Whatman 903) from TIHS, Australia, dating more than 20 years old, were also used to test the efficiency of the robust protocol GQ. Both NCS and TIHS samples were heel-prick without anticoagulants added. Permissions from the ethical committees of the International Agency for Research on Cancer (IARC), as well as both, NCS and TIHS, were obtained. TIHS is one of the founder cohorts of the International Childhood Cancer Cohort Consortium (I4C) [1].

### DNA extraction protocols

Combinations of different commercially available DNA extraction kits were used, including QIAamp DNA Micro Kit (Qiagen 56304), GenSolve (Gen Vault, GVR110), NucleoSpin (gDNA clean-up, Macherey-Nagel 740230), and the extra-small (XS) version of NucleoSpin (gDNA clean-up XS, Macherey-Nagel 740904). Reported quantifications were done using Nanodrop, unless indicated otherwise using Qubit™ dsDNA High Sensitivity Assay, Invitrogen Q32851. Detailed protocols are included in Additional Methods (Additional File 2).

### Gel electrophoresis and bioanalyzer analysis

Samples were run on a 0.8% agarose gel (Eurobio GEPAGA07-65) in 1 × Tris Acetate-EDTA buffer and stained with GelRed. 300 ng of DBS DNA were utilized per sample in electrophoresis analyses of DNA integrity and size range. The following DNA size markers were used: 80–10,000 bp ladder (Thermo SM0403), 500–5,000 bp ladder (Takara 3411A), and 100–1,000 bp ladder (Thermo SM0243). As for bioanalyzer analysis, 500 pg of DNA per sample was loaded on the chip and analyzed on Agilent 2100 Bioanalyzer, as per manufacturer’s instructions (Agilent Technology, High Sensitivity DNA Kit, 5067–4626).

### Bisulfite conversion and PCR

DBS DNA (300 ng) samples were bisulfite converted using EZ DNA Methylation Kit (Zymo Research D5001) according to manufacturer’s instructions. To assess the efficiency of bisulfite conversion, DNA was amplified using PCR primers that were specific either to bisulfite-converted or to non-modified DNA, and spanning the region of the housekeeping gene, *GAPDH*. The primer pairs spanning bisulfite-converted *GAPDH* regions are termed GAPDH-bc and consisted of the following forward and reverse primers, respectively: 5’-GTATTTGTTGATGGGTTAAGG-3’ and 5’-ATAAAAACAAATCCCCTACCC-3’. The primer pairs spanning non-modified *GAPDH* regions are termed GAPDH-nm and consisted of the following forward and reverse primers, respectively: 5’-CTCTTGCTACTCTGCTCTGG-3’ and 5’-GCTAAGTTTAGCCTGCCTGG-3’. Efficient conversion is observed when PCR bands are detected with GAPDH-bc but not with GAPDH-nm for a given sample. The PCR conditions used were: 95°C 15 min, [95°C 30 s, 57°C 30 s, 72°C 30 s] × 50 cycles, 72°C 10 min, and pause 4°C.

### Pyrosequencing

DNA methylation analysis by pyrosequencing of bisulfie-converted DNA was performed as described [22]. Briefly, the region of interest was amplified with forward and reverse primers, one of which is biotinylated (btn), and then the methylation levels of the amplified region were analyzed using a sequencing primer. The forward, reverse and sequencing primers used for Line1 were the following, respectively, and adopted from Daskalos et al. [16]: 5’-btn-TAGGGAGTGTTAGATAGTGG-3’, 5’-AACTCCCTAACCCCTTAC-3’ and 5’-CAAATAAAACAATACCTC-3’. The forward, reverse and sequencing primers used for AluYb8 were the following, respectively: 5’-AGATTATTTTGGTTAATAAG-3’, 5’-btn-AACTACRAACTACAATAAC-3’ and 5’-GTTTGTAGTTTTAGTTATT- 3’, as previously described [23].

### Illumina Infinium HM450 array and data processing

Infinium HM450 arrays were processed according to manufacturer’s instructions. GenomeStudio was used to analyze quality controls. The raw colour channels were corrected using the internal control probes and converted, without background subtraction and normalization, into absolute methylation levels (beta-values). Data were then imported into R (3.0.0), using the *minfi* package version 1.2.0 (http://www.bioconductor.org). Subset-quantile Within Array Normalisation (SWAN) normalisation was performed to correct for technical discrepancies between Type I and Type II [24]. Probes with detection p-values above 0.01 were considered as background noise and omitted from further analysis. Sex chromosome-specific probes were eliminated to minimize gender-specific variation of the X *versus* Y chromosomes [25]. Logarithmic transformation of the beta-values into M values was done, as previously described [26]. Statistical tests were performed using M-values. Clustering plots were generated using the *lumi* R package and based on coefficient of variation, which is calculated by standard deviation divided by mean across samples [27]. Differentially methylated CpGs were identified using an F-test in *minfi*.

## Abbreviations

bp: base pair; DBS: dried blood spots; gDNA: genomic DNA; GQ: GenSolve-QIAamp; GN: GenSolve-NucleoSpin; Gn: GN with NucleoSpin’s precipitation buffer replaced by ethanol; GN-XS: GN extra small; Gn-XS: GN-XS with NucleoSpin’s precipitation buffer replaced by ethanol; HM450: Infinium HumanMethylation450 Beadchip; Kbp: kilo base pair; NCS: National Children Study - USA; NN: NucleoSpin-NucleoSpin; Nn: NN with NucleoSpin’s precipitation buffer replaced by ethanol; NN-XS: NN extra small; QC: quality control; QQ: QIAamp-QIAamp; Qq: QQ with QIAamp’s precipitation buffer replaced by ethanol; SWAN: Subset-quantile Within Array Normalisation; TIHS: Tasmanian Infant Health Survey - Australia.

## Competing interests

No conflicts of interest are disclosed.

## Authors’ contributions

AG carried out all experiments and drafted the manuscript. MPC participated in the optimization of the protocols. ALP participated in the collection of TIHS samples. SH and KC participated in the collection of NCS samples. ZH, TD, RS and HH conceived the study and participated in its design and coordination and helped to draft the manuscript. AG and HH performed the statistical and bioinformatics analyses. All authors read and approved the final manuscript.

## Supplementary Material

Additional file 1 Whole bisulfitome amplification. Click here for file

Additional file 2 Additional Methods. Click here for file

Additional file 3 Infinium HM450 quality control probes (minfi package). Click here for file

Additional file 4 Correlations between technical replicates. Click here for file
