# Supplementary figures and images for: Optimized DNA extraction from neonatal dried blood spots: application in methylome profiling
Source: BMC Biotechnol. 2014 Jul 1;14:60. doi: 10.1186/1472-6750-14-60 (PMC4086704; doi:10.1186/1472-6750-14-60)

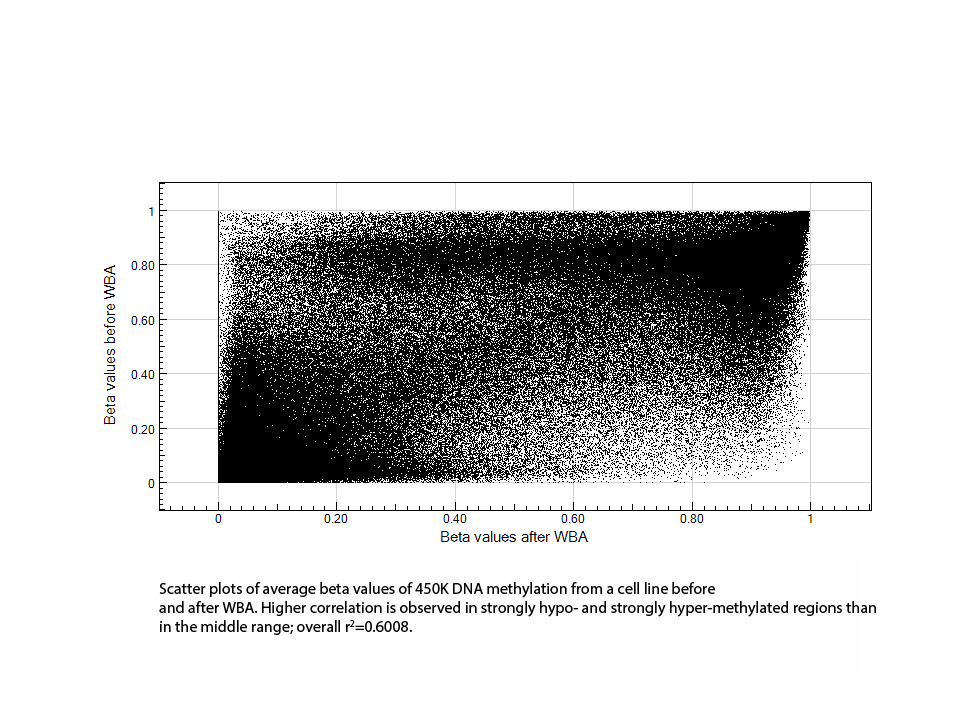

Supplement: Additional file 1 — Whole bisulfitome amplification. [file 1472-6750-14-60-S1.tiff]

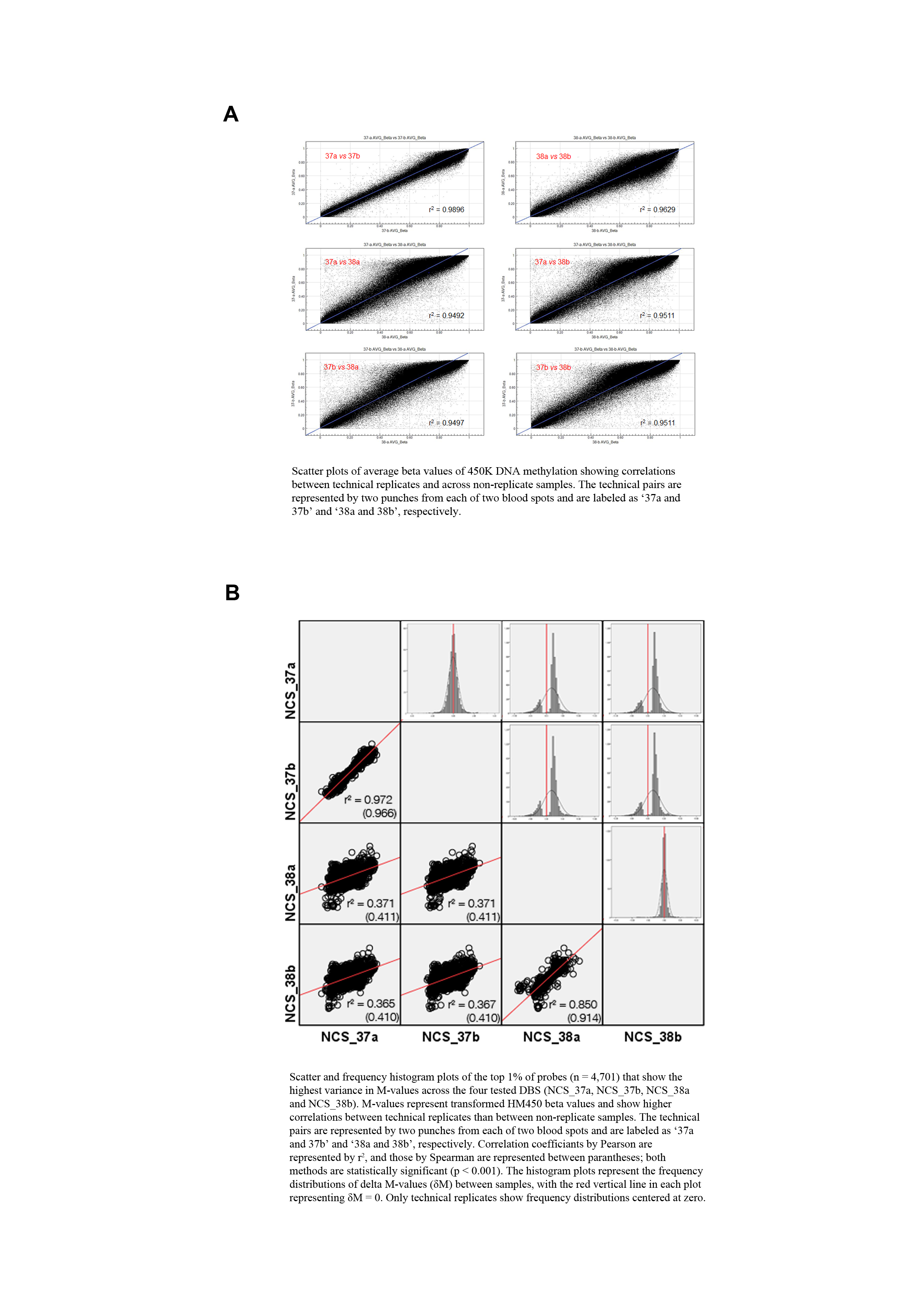

Supplement: Additional file 4 — Correlations between technical replicates. [file 1472-6750-14-60-S4.tiff]
