# Supplementary material for: Optimized DNA extraction from neonatal dried blood spots: application in methylome profiling
Source: BMC Biotechnol. 2014 Jul 1;14:60. doi: 10.1186/1472-6750-14-60 (PMC4086704; doi:10.1186/1472-6750-14-60)
Supplement: Additional file 2 — Additional Methods. [file 1472-6750-14-60-S2.docx]

**Supplementary Methods**

The following DNA extraction protocols are described for a DBS punch having a 9 mm diameter (equivalent to 4 punches with a 4.5 mm diameter each and to a total area of 63.6 mm^2^). The provided solution volumes may be tailored proportionally to the DBS area in use, although the volume provided for the starting solution, the lysis-protease mix, is given in excess to ensure complete soaking of DBS pieces and was set at 620 µl for all protocols. Centrifugation speeds for elution were also set constant across protocols. Use sterile equipment and avoid cross-contamination between samples by cleaning equipment with deionized water followed by 70% ethanol after each use. Cut the DBS punches into small pieces using a sterile scissors, over a clean filter paper, and transfer the pieces into a 2 ml microtube (replace the filter paper between samples). The protocols described next proceed afterwards, using the microtube containing the DBS pieces. The DNA yield (ng) was obtained by multiplying the DNA concentration prior to heating by the measured eluted volume. Reported quantifications were done using Nanodrop, unless indicated otherwise. Nanodrop performance was ascertained using two standards, 10 ng/μl and 100 ng/μl, with a maximum of 3.5% error and with r^2^=0.999 over a standard range of 0, 2.5, 5 and 10 ng/μl. The following suggestions apply to all the tested protocols:

1. The following steps are recommended to fully extract blood material from filter papers: soaking DBS in large lysis buffer volumes (620 µl), incubation in lysis solution for extended durations (1.5-3 h) with increased shaking speeds (1,400 rpm) and using spin baskets (Fig. 1A) at high speed. TissueLyser (Qiagen) no longer increases the DNA yield above that obtained using these modifications (data not shown).
2. The protease and lysis solutions in Phase I may be proportionally mixed according to each protocol before being added to each microtube sample. However, it is recommended to add the mixture to the samples immediately after its preparation because the protease may otherwise self-digest in the absence of substrate.
3. Ethanol, unlike many buffers tested to precipitate DNA, causes less precipitates in lysed DBS solution, so prolonged vortexing is not necessary to ensure homogenization and should be replaced by inversion mixing after ethanol addition to prevent DNA fragmentation.
4. After binding DNA to the spin column and adding each wash buffer, turn the column inside the centrifuge by 180° compared to the loading position used for binding because this allows optimal washing efficiency.
5. With spin columns, two sequential elutions using the same volume, X µl, of eluent in the same column generally yields more DNA than using 2X µl of eluent in one shot. However, this would not be the case if X is less than the minimum elution capacity of the column because then a single elution with X may not be sufficient to completely soak the column resin.
6. Preheating the nuclease-free water (or elution buffer) at 70 °C before elution and then incubating the elution column (containing preheated water) at 70 °C for 10 min increases the DNA yield. This is because DNA bound to spin columns dissolves better in warmer solution; hence, it is eluted in higher quantities.
7. Heating the DNA eluates at 65 °C will concentrate the DNA allowing longer storage durations for a given yield, will not denature DNA but can denature enzymes that may degrade DNA, and will cause the evaporation of volatile buffer contaminants, including ethanol, which may mess up the 260/280 and 260/280 ratios and/or inhibit many subsequent amplification reactions, including PCR. Shaking the eluates while heating minimizes, in a speed-dependent manner, the incubation time necessary to concentrate the samples.
8. All centrifugation steps are carried out at room temperature (15-25 °C).

Protocol QQ:

The following steps were adopted and modified from the QIAamp DNA Micro kit (Qiagen 56304) with modifications.

*Phase I (use QIAamp reagents):*

1. Add 620 µl of lysis-protease mix, which is composed of 560 µl Buffer ATL (Qiagen 56304) + 60 µl proteinase K (Qiagen 56304), per microtube containing the DBS pieces.
2. Incubate at 56 °C with shaking at 1,400 rpm for 1.5 h (inspect during the first 5 min that all pieces are completely submerged).
3. Briefly spin to collect liquid off cap.
4. Transfer the solution from step 3 into an empty spin basket (GVSPIN250), making sure to transfer along the DBS pieces by scooping them with a pipette tip.
5. Spin at 16,300 x g for 2 min. Discard basket and paper pieces. Pulse vortex each tube.

*Phase II (use QIAamp reagents):*

1. Add 600 µl of Buffer AL (Qiagen 56304) per sample.  A top layer of white precipitates forms, so vortex tubes for 10 s to enhance dissolution, briefly spin the tubes and incubate them at 70 °C for 10 min with shaking at 900 rpm to allow complete dissolution. Pulse vortex then briefly spin afterwards to collect liquid from the caps.
2. Load 600 µl of the sample onto a QIAamp spin column/collection tube (Qiagen 56304). Spin at 6,000 x g for 1 min. Discard the eluate and tap the top of the collection tube on clean tissue.
3. Repeat step 7 until the entire sample has been applied onto the column.
4. Add 25 ml ethanol (96-100 %) to the bottle containing 19 ml Buffer AW1 (Qiagen 56304); mix by shaking. Reconstituted AW1 can be stored at room temperature for up to 1 year. Add 500 µl of AW1 onto each column and spin at 6,000 x g for 1 min. Discard the eluate and tap the collection tube on tissue.
5. Add 30 ml ethanol (96-100 %) to the bottle containing 13 ml Buffer AW2 (Qiagen 56304); mix by shaking. Reconstituted AW2 can be stored at room temperature for up to 1 year. Add 500 µl AW2 onto each column and spin at 16,300 x g for 4 min. Discard collection tube and replace with a new one.
6. Spin again at 16,300 x g for 1.5 min.
7. Place the column in a new 1.7 ml tube.
8. Add 50 µl of nuclease-free water (preheated at 70 °C) to the spin column to elute DNA. Incubate at room temperature for 5 min. Spin at 6,000 x g for 1 min. Use 1 µl of the eluate in PCR to verify the presence of DNA amplicons.
9. Quantitate DNA concentrations (we used Nanodrop, using nuclease-free water as a blank, and Qubit™ dsDNA High Sensitivity Assay, Invitrogen Q32851).
10. Open the covers of the microtubes containing the DNA eluates, place the tubes in a heat block, cover them with an aluminum foil to prevent contamination by dust, and heat them at 65 °C for at least 10 min (65 min were used on our samples). Store at -20 °C.

Protocol Qq:

*Phase I (use QIAamp reagents):*

1. Proceed with steps 1-5, as in protocol QQ.

*Phase II (use QIAamp reagents):*

1. Add 600 µl of 100 % ethanol to each microtube (replacing precipitation buffer AL of QQ; AL, unlike ethanol, causes flocks of solid precipitates that might have hindered the later steps of DNA purification, leading to decreased DNA yield in QQ). Pulse-vortex each sample (avoid excessive vortexing which may cause DNA fragmentation). Heating to homogenize the solution, as in QQ, is not necessary here. Briefly spin.
2. Proceed with steps 7-15, as in protocol QQ.

Protocol GQ:

The following steps were adopted and modified from the GenSolve (GenVault, GVR110) and QIAamp DNA Micro (Qiagen 56304) kits.

*Phase I (use GenSolve reagents):*

1. Reconstitute Solution A powder (GVR110) with 16 ml of 1 % LiDs (GVR110) to form the lysis solution. Vortex to completely resuspend the lyophilized reagent. The reconstituted solution can be stored for at least one month at + 4 °C without loss of lysis potential (Table S1). Add 620 µl of lysis-protease mix, which is composed of 600 µl of reconstituted Solution A + 20 µl protease (GVR110), per microtube containing the DBS pieces.
2. Incubate at 65 °C with shaking at 1,400 rpm for 1.5 h (inspect during the first 5 min that all pieces are completely submerged).
3. Briefly spin to collect liquid off cap.
4. Add 20 µl Recovery Solution B (GVR110) to an empty spin basket (GVSPIN250) and then transfer the solution from step 3 into the basket, making sure to transfer along the DBS pieces by scooping them with a pipette tip.
5. Spin at 16,300 x g for 2 min. Discard basket and paper pieces. Pulse vortex each tube.

*Phase II (use QIAamp reagents):*

1. Add 600 µl of 100 % ethanol to each microtube. Pulse-vortex each sample (avoid excessive vortexing which may cause DNA fragmentation). Briefly spin.
2. Proceed with steps 7-15, as in protocol QQ.

Protocol GN:

The following steps were adopted and modified from the GenSolve (GenVault, GVR110) and NucleoSpin gDNA Clean-up (Macherey-Nagel 740230) kits.

*Phase I (use GenSolve reagents):*

1. Proceed with steps 1-5, as in protocol GQ.

*Phase II (use NucleoSpin reagents):*

6. Add 1,660 µl of Binding Buffer DB (Macherey-Nagel 740230) to each microtube. Vortex for 5 s.

7. Load 700 µl of the sample onto a gDNA Clean-up column/collection tube (Macherey-Nagel 740230). Spin at 11,000 x g for 0.5 min. Discard the eluate and tap the top of the collection tube on clean tissue.

8. Repeat step 7 until the entire sample has been applied onto the column.

9. Add 14 ml ethanol (96-100 %) to the bottle containing 6 ml Wash Buffer DW (Macherey-Nagel 740230.10); mix by shaking. Reconstituted DW can be stored at room temperature for up to 1 year. Add 700 µl of DW onto each column, vortex for 2 s and spin at 11,000 x g for 0.5 min. Discard the eluate and tap the collection tube on tissue.

10. Repeat the wash step above

11. Spin again at 11,000 x g for 1 min.

12. Proceed with steps 12-15, as in protocol QQ.

Protocol Gn:

*Phase I (use GenSolve reagents):*

1. Proceed with steps 1-5, as in protocol GQ.

*Phase II (use NucleoSpin reagents):*

1. Add 600 µl of 100 % ethanol to each microtube (replacing precipitation buffer DB of GN). Pulse-vortex each sample (avoid excessive vortexing which may cause DNA fragmentation). Briefly spin.
2. Proceed with steps 7-15, as in protocol GN.

Protocol GN-XS:

The following steps were adopted and modified from the GenSolve (GenVault, GVR110) and NucleoSpin gDNA Clean-up XS (Macherey-Nagel 740904) kits.

*Phase I (use GenSolve reagents):*

1. Proceed with steps 1-5, as in protocol GQ.

*Phase II (use NucleoSpin reagents):*

6. Add 150 µl of Buffer NT (Macherey-Nagel 740904) to each microtube. Vortex for 5 s.

7. Load 500 µl of the sample onto a gDNA Clean-up XS column/collection tube (Macherey-Nagel 740904). Spin at 11,000 x g for 0.5 min. Discard the eluate and tap the top of the collection tube on clean tissue.

8. Repeat step 7 until the entire sample has been applied onto the column.

9. Add 24 ml ethanol (96-100 %) to the bottle containing 6 ml Wash Buffer B5 (Macherey-Nagel 740904.10); mix by shaking. Reconstituted B5 can be stored at room temperature for up to 1 year. Add 100 µl of B5 onto each column and spin at 11,000 x g for 2 min.

10. Proceed with steps 12-15, as in protocol QQ.

Protocol Gn-XS:

*Phase I (use GenSolve reagents):*

1. Proceed with steps 1-5, as in protocol GQ.

*Phase II (use NucleoSpin reagents):*

1. Add 600 µl of 100 % ethanol to each microtube (replacing precipitation buffer NT of GN-XS). Pulse-vortex each sample (avoid excessive vortexing which may cause DNA fragmentation). Briefly spin.
2. Proceed with steps 7-10, as in protocol GN-XS.

Protocol NN:

The following steps were adopted and modified from the NucleoSpin Tissue kit (Macherey-Nagel 740952).

*Phase I (use NucleoSpin reagents):*

1. Add 620 µl of lysis-protease mix, which is composed of 544 µl Buffer T1 (Macherey-Nagel 740952) + 60 µl proteinase K (Macherey-Nagel 740952), per microtube containing the DBS pieces.
2. Proceed with steps 2-5, as in protocol QQ.

*Phase II (use NucleoSpin reagents):*

1. Add 600 µl of Buffer B3 (Macherey-Nagel 740952) to each microtube. A top layer of precipitates forms, so vortex tubes for 10 s to enhance dissolution, briefly spin the tubes and incubate them at 70 °C for 10 min to allow complete dissolution. Pulse vortex then briefly spin afterwards to collect liquid from the caps. Add 630 µl ethanol (96-100 %) to the sample, vortex for 10 s and then briefly spin.
2. Load 600 µl of the sample onto a NucleoSpin Tissue column/collection tube (Macherey-Nagel 740952). Spin at 11,000 x g for 1 min. Discard the eluate and tap the top of the collection tube on clean tissue.
3. Repeat step 7 until the entire sample has been applied onto the column.
4. Add 500 µl of Buffer BW (Macherey-Nagel 740952.10) onto each column and spin at 11,000 x g for 1 min. Discard the eluate and tap the collection tube on tissue.
5. Add 16 ml ethanol (96-100 %) to the bottle containing 4 ml Wash Buffer B5 (Macherey-Nagel 740952.10); mix by shaking. Reconstituted B5 can be stored at room temperature for up to 1 year. Add 600 µl of B5 onto each column and spin at 11,000 x g for 1 min. Discard collection tube and replace with a new one.
6. Spin again at 11,000 x g for 1 min.
7. Proceed with steps 12-15, as in protocol QQ.

Protocol Nn:

*Phase I (use NucleoSpin reagents):*

1. Proceed with steps 1-5, as in protocol NN.

*Phase II (use NucleoSpin reagents):*

1. Add 600 µl of 100 % ethanol to each microtube (replacing precipitation buffer B3 of NN). Pulse-vortex each sample (avoid excessive vortexing which may cause DNA fragmentation). Briefly spin.
2. Proceed with steps 7-12, as in protocol NN.

Protocol NN-XS:

The following steps were adopted and modified from the NucleoSpin Tissue XS kit[^12^](#_ENREF_12) (Macherey-Nagel 740901).

*Phase I (use NucleoSpin reagents):*

1. Add 620 µl of lysis-protease mix, which is composed of 564 µl Buffer T1 (Macherey-Nagel 740901) + 196 µl proteinase K (Macherey-Nagel 740901), per microtube containing the DBS pieces.
2. Proceed with steps 2-5, as in protocol QQ.

*Phase II (use NucleoSpin reagents):*

1. Add 600 µl of Buffer B3 (Macherey-Nagel 740901) to each microtube. A top layer of precipitates forms, so vortex tubes for 10 s to enhance dissolution, briefly spin the tubes and incubate them at 70 °C for 10 min to allow complete dissolution. Pulse vortex then briefly spin afterwards to collect liquid from the caps. Add 600 µl ethanol (96-100 %) to the sample, vortex for 10 s and then briefly spin.
2. Load 500 µl of the sample onto a NucleoSpin Tissue XS column/collection tube (Macherey-Nagel 740901). Spin at 11,000 x g for 1 min. Discard the eluate and tap the top of the collection tube on clean tissue.
3. Repeat step 7 until the entire sample has been applied onto the column.
4. Add 4 ml ethanol (96-100 %) to the bottle containing 1 ml Wash Buffer B5 (Macherey-Nagel 740901.10); mix by shaking. Reconstituted B5 can be stored at room temperature for up to 1 year. Add 50 µl of B5 onto each column and spin at 11,000 x g for 1 min.
5. Add 50 µl of B5 onto each column and spin at 11,000 x g for 2 min. Discard collection tube.
6. Proceed with steps 12-15, as in protocol QQ.
