# Supplementary material for: Optimized DNA extraction from neonatal dried blood spots: application in methylome profiling
Source: BMC Biotechnol. 2014 Jul 1;14:60. doi: 10.1186/1472-6750-14-60 (PMC4086704; doi:10.1186/1472-6750-14-60)
Supplement: Additional file 3 — Infinium HM450 quality control probes (minfi package). [file 1472-6750-14-60-S3.pdf]

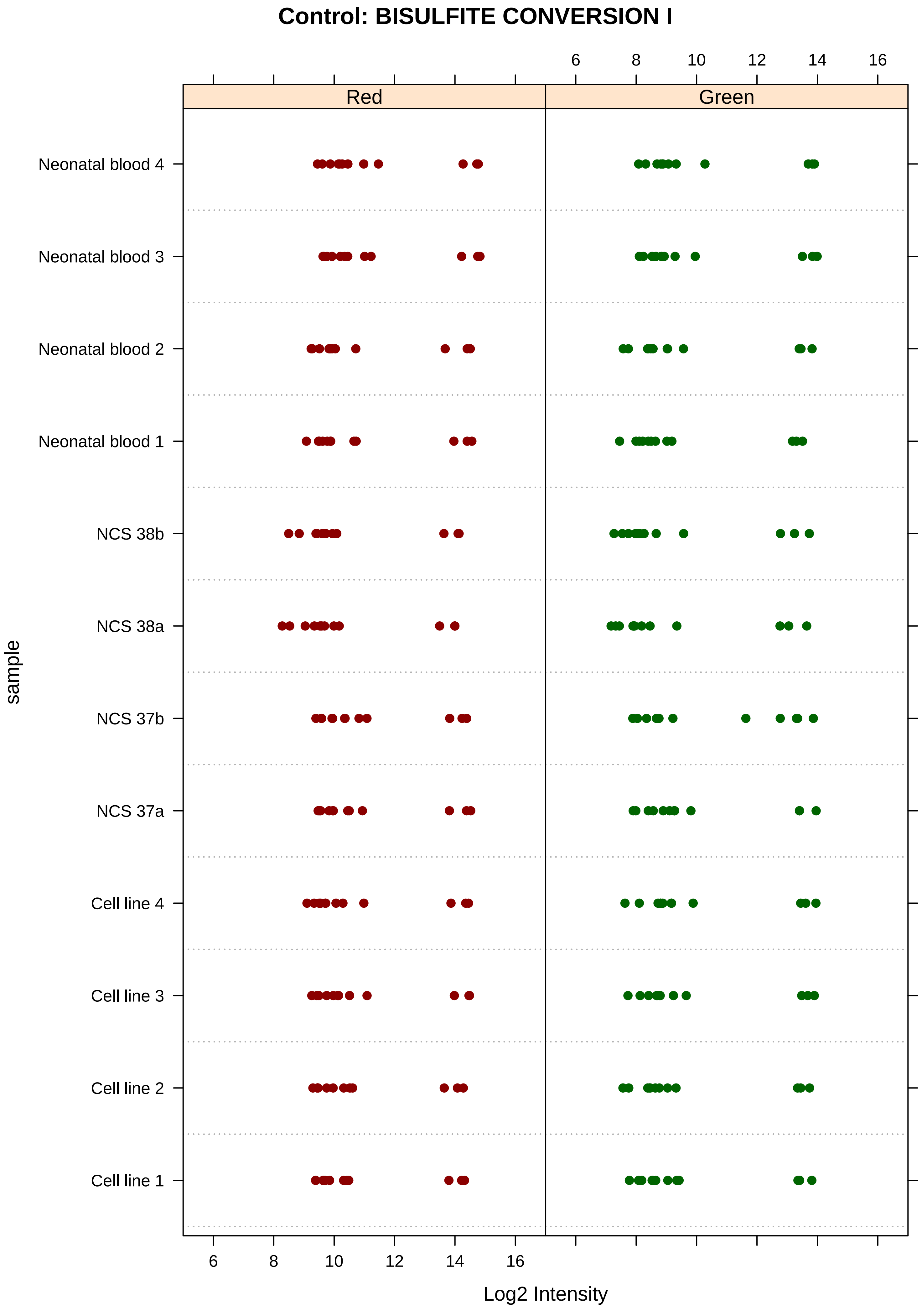
Control: BISULFITE CONVERSION I
Red
Green
Neonatal blood 4
Neonatal blood 3
Neonatal blood 2
Neonatal blood 1
NCS 38b
NCS 38a
NCS 37b
NCS 37a
Cell line 4
Cell line 3
Cell line 2
Cell line 1
sample
6
8
10
12
14
16
Log2 Intensity

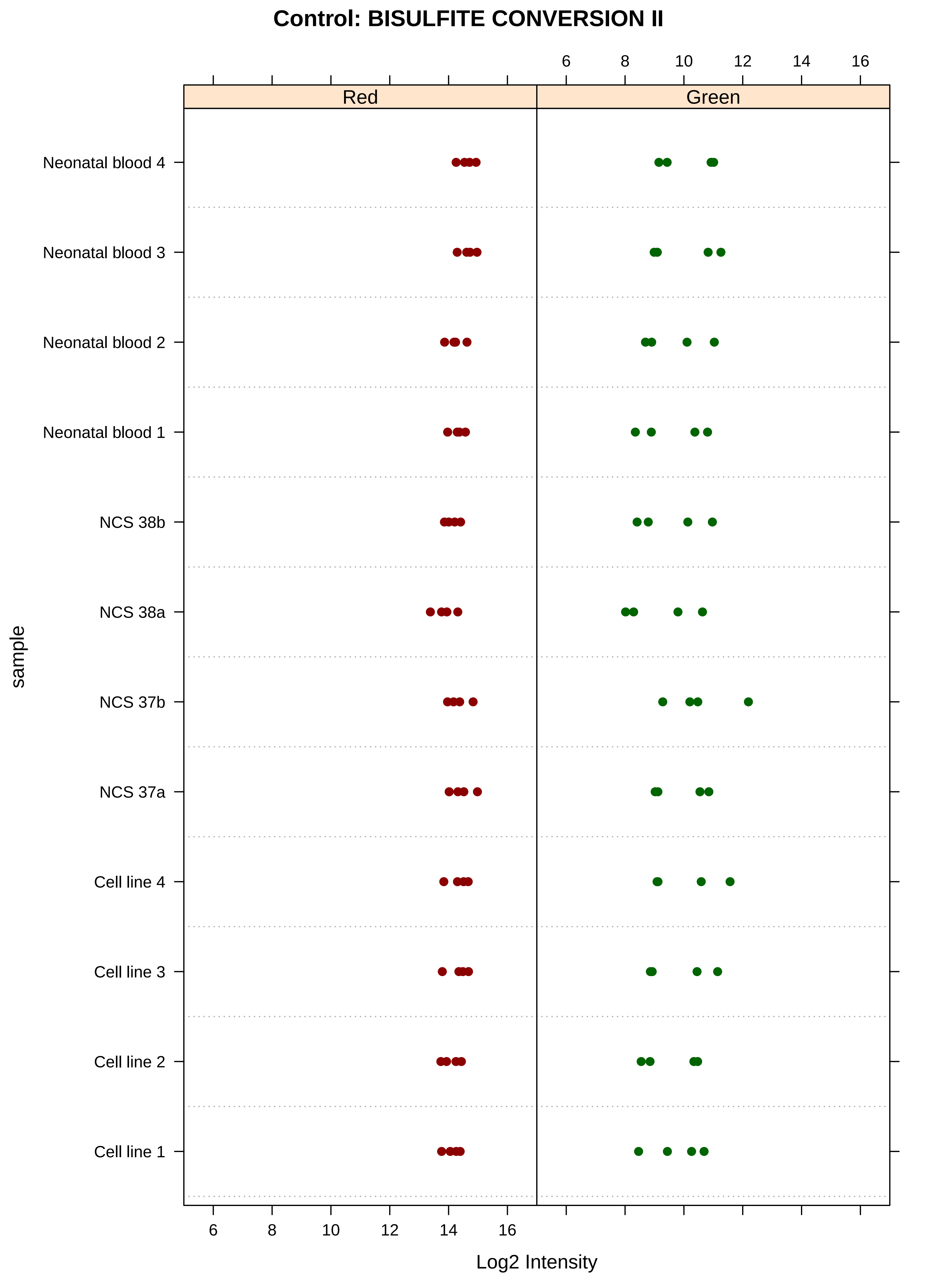

Control: BISULFITE CONVERSION II
Red
Green
Neonatal blood 4
Neonatal blood 3
Neonatal blood 2
Neonatal blood 1
NCS 38b
NCS 38a
NCS 37b
NCS 37a
Cell line 4
Cell line 3
Cell line 2
Cell line 1
sample
6
8
10
12
14
16
Log2 Intensity

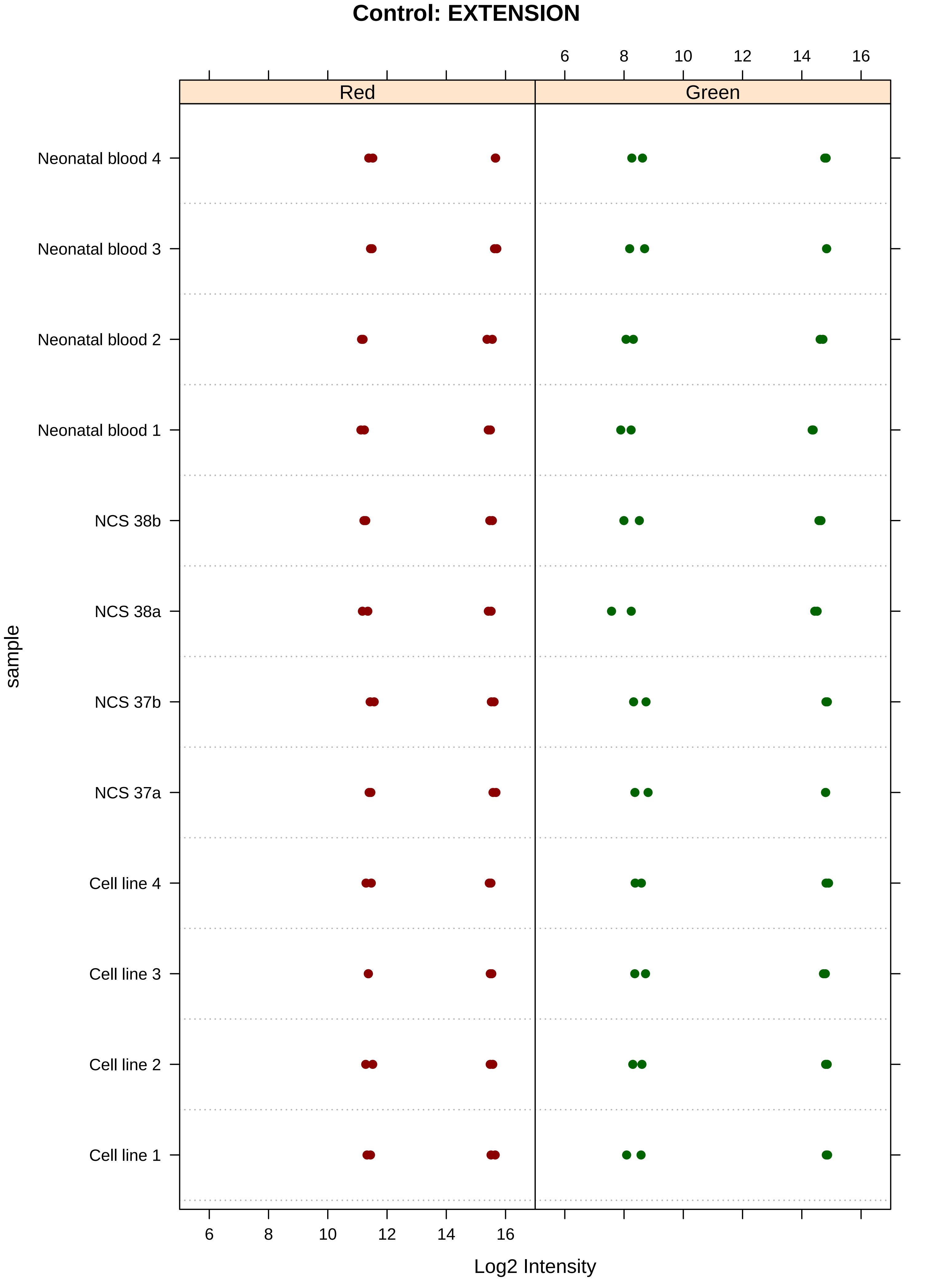

Control: EXTENSION
Red
Green
Neonatal blood 4
Neonatal blood 3
Neonatal blood 2
Neonatal blood 1
NCS 38b
NCS 38a
NCS 37b
NCS 37a
Cell line 4
Cell line 3
Cell line 2
Cell line 1
sample
6
8
10
12
14
16
Log2 Intensity

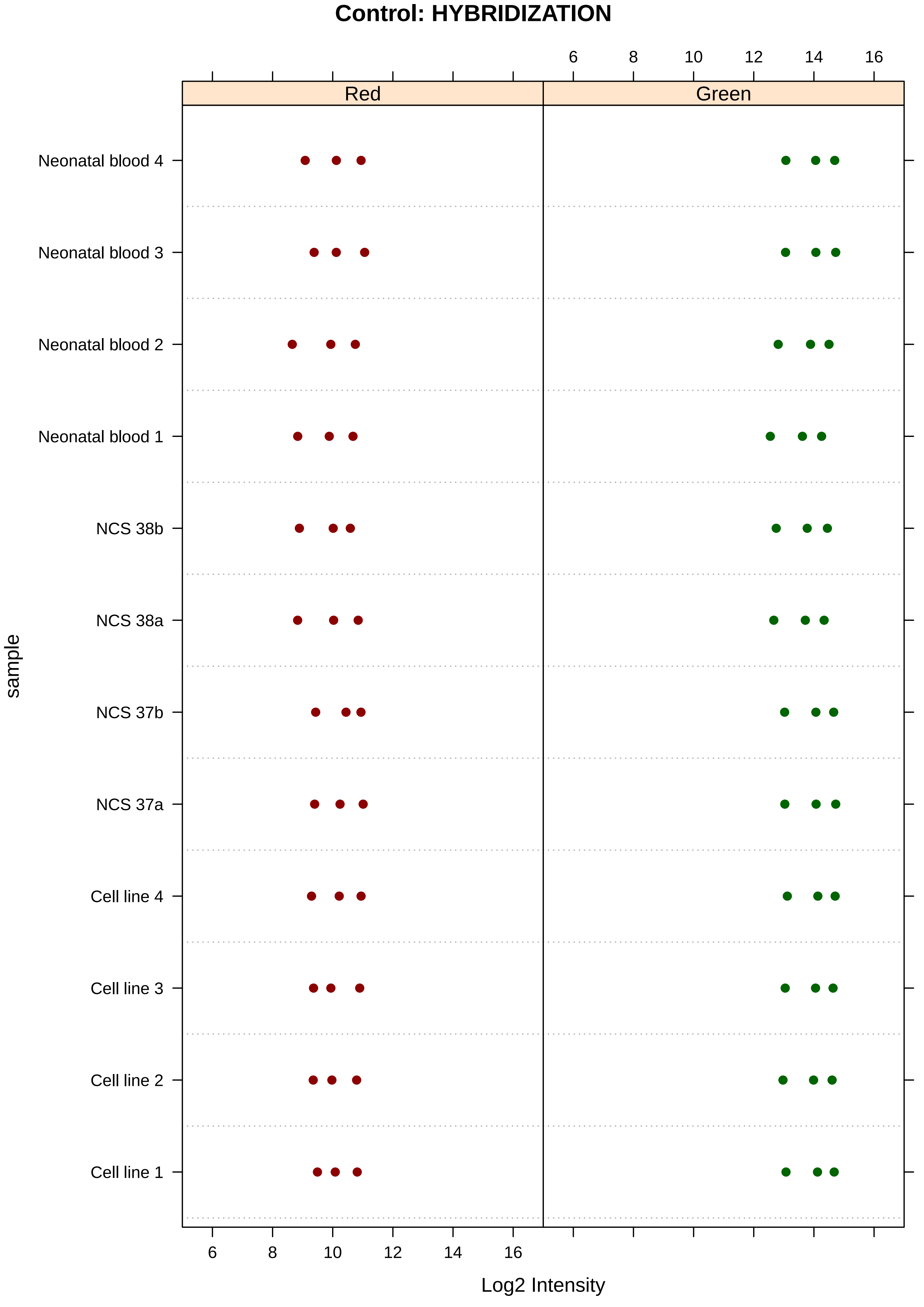

Control: HYBRIDIZATION
Red
Green
Neonatal blood 4
Neonatal blood 3
Neonatal blood 2
Neonatal blood 1
NCS 38b
NCS 38a
NCS 37b
NCS 37a
Cell line 4
Cell line 3
Cell line 2
Cell line 1
sample
6
8
10
12
14
16
Log2 Intensity

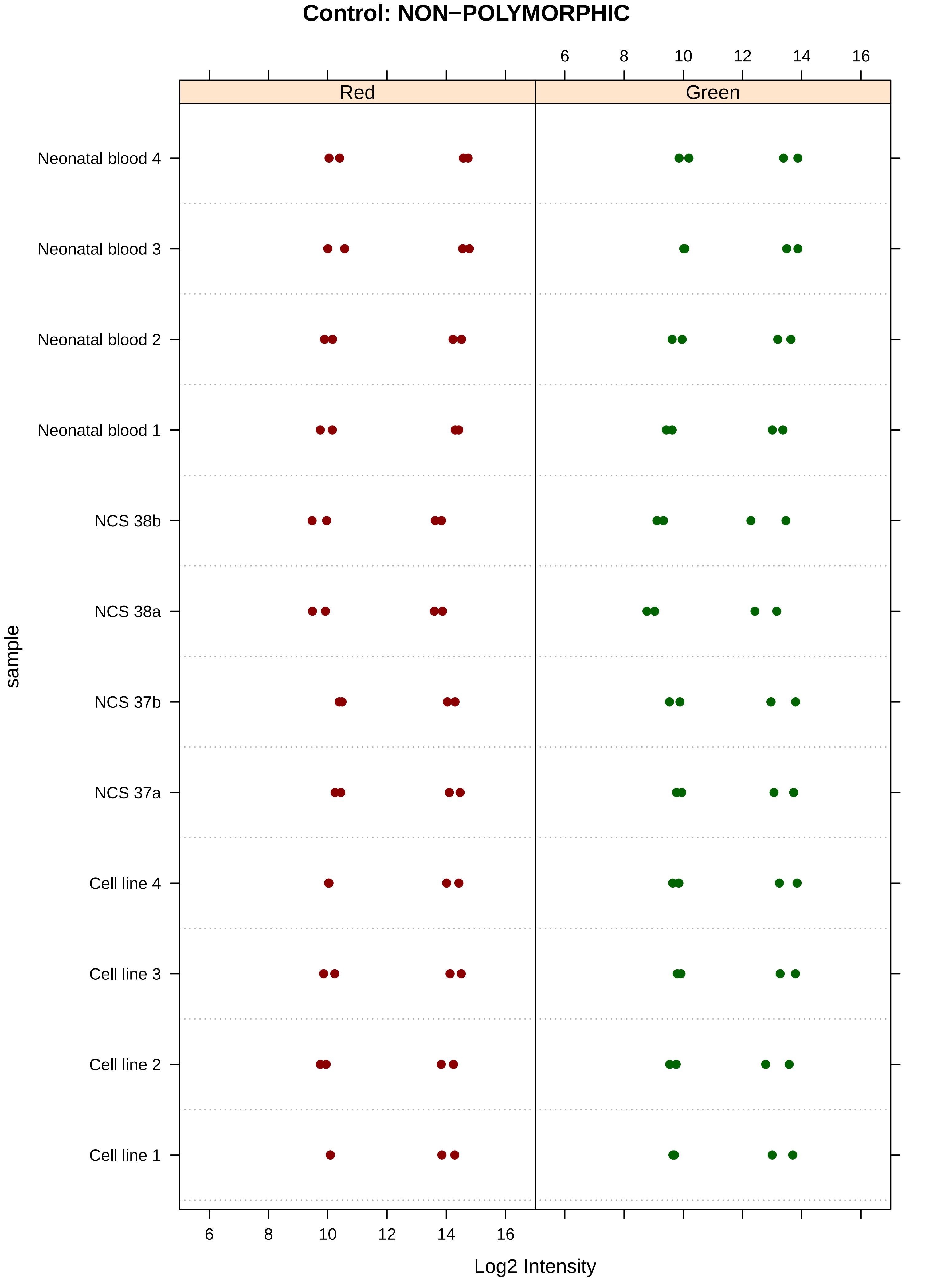

Control: NON−POLYMORPHIC
Red
Green
Neonatal blood 4
Neonatal blood 3
Neonatal blood 2
Neonatal blood 1
NCS 38b
NCS 38a
NCS 37b
NCS 37a
Cell line 4
Cell line 3
Cell line 2
Cell line 1
sample
6
8
10
12
14
16
Log2 Intensity

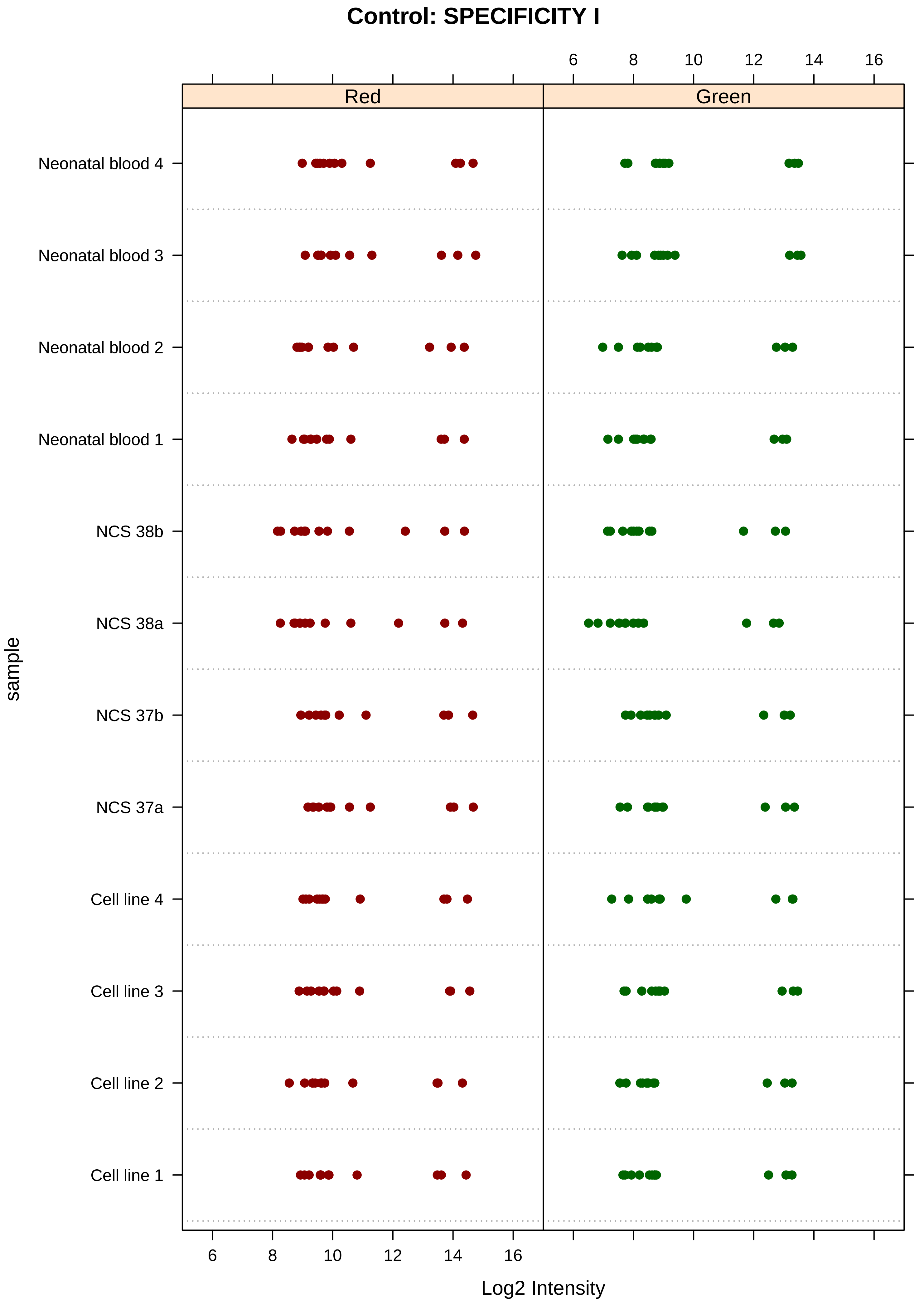

Control: SPECIFICITY I
Red
Green
Neonatal blood 4
Neonatal blood 3
Neonatal blood 2
Neonatal blood 1
NCS 38b
NCS 38a
NCS 37b
NCS 37a
Cell line 4
Cell line 3
Cell line 2
Cell line 1
sample
6
8
10
12
14
16
Log2 Intensity

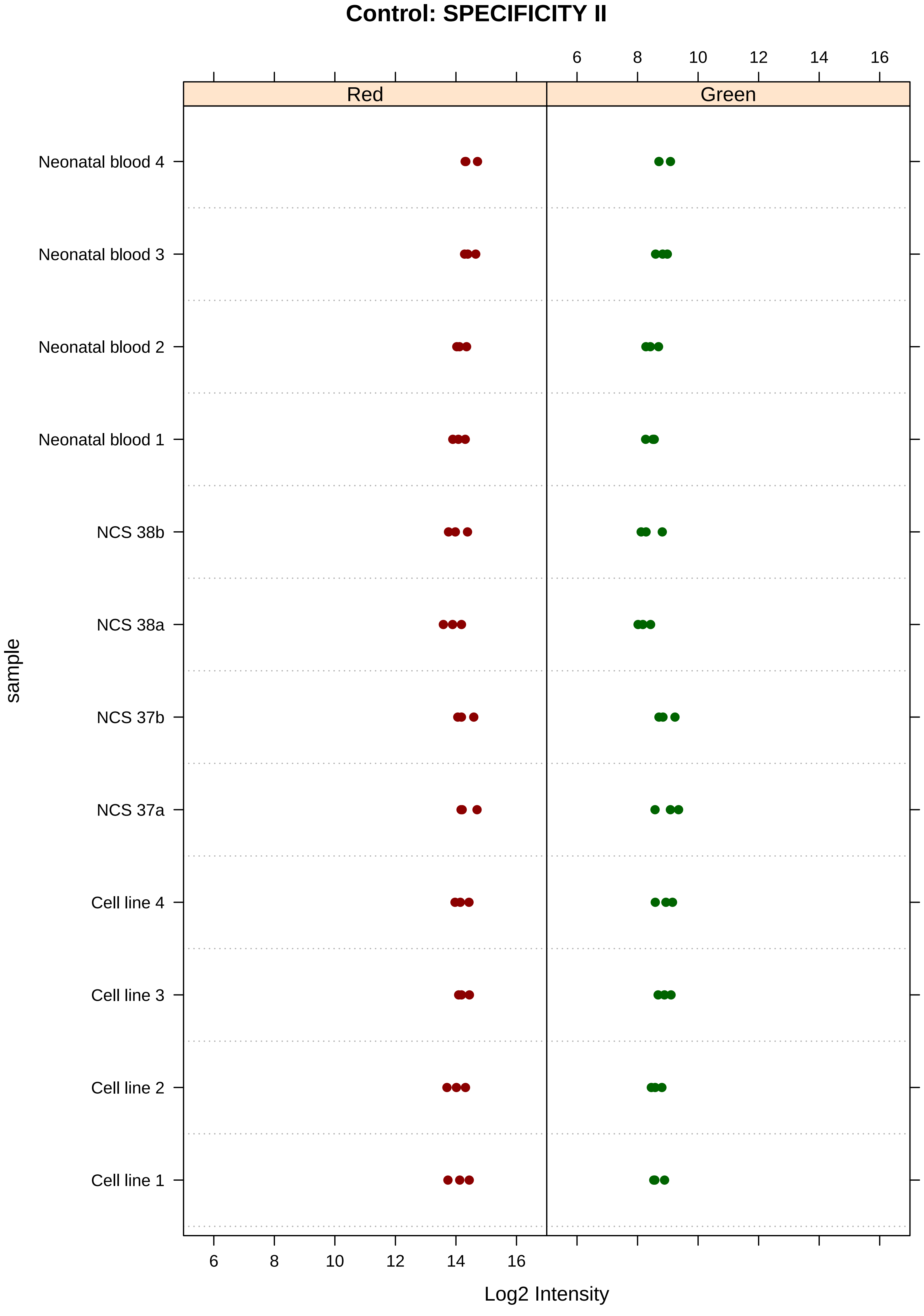

Control: SPECIFICITY II
Red
Green
Neonatal blood 4
Neonatal blood 3
Neonatal blood 2
Neonatal blood 1
NCS 38b
NCS 38a
NCS 37b
NCS 37a
Cell line 4
Cell line 3
Cell line 2
Cell line 1
sample
6
8
10
12
14
16
Log2 Intensity

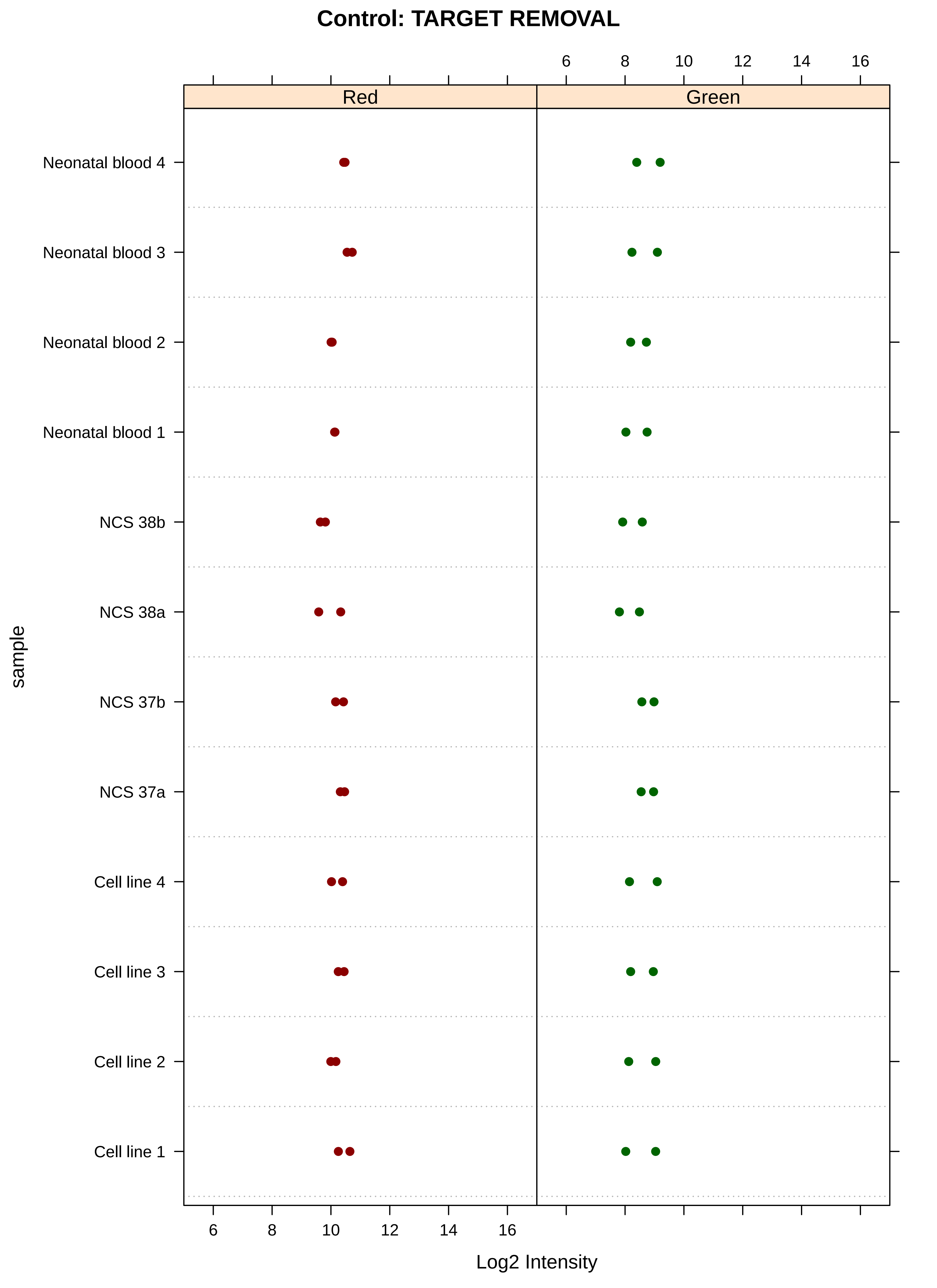

Control: TARGET REMOVAL
Red
Green
Neonatal blood 4
Neonatal blood 3
Neonatal blood 2
Neonatal blood 1
NCS 38b
NCS 38a
NCS 37b
NCS 37a
Cell line 4
Cell line 3
Cell line 2
Cell line 1
sample
6
8
10
12
14
16
Log2 Intensity
